# Drug-Targeted Genomes: Mutability of Ion Channels and GPCRs

**DOI:** 10.3390/biomedicines10030594

**Published:** 2022-03-03

**Authors:** Regan Raines, Ian McKnight, Hunter White, Kaitlyn Legg, Chan Lee, Wei Li, Peter H. U. Lee, Joon W. Shim

**Affiliations:** 1 Department of Biomedical Engineering, College of Engineering and Computer Sciences, Marshall University, Huntington, WV 25755, USA; raines83@marshall.edu (R.R.); mcknight31@marshall.edu (I.M.); white869@marshall.edu (H.W.); legg144@marshall.edu (K.L.); 2 Indiana University Health Arnett Hospital, Lafayette, IN 47905, USA; clee9@iuhealth.org; 3 Department of Biomedical Sciences, Joan C. Edwards School of Medicine, Marshall University, Huntington, WV 25755, USA; liwe@marshall.edu; 4 Department of Pathology and Laboratory Medicine, Brown University, Providence, RI 02912, USA; peter_lee@brown.edu; 5 Department of Cardiothoracic Surgery, Southcoast Health, Fall River, MA 02720, USA

**Keywords:** target therapy, ion channels, GPCRs, telomere, mutation, cardiovascular pharmacology

## Abstract

Mutations of ion channels and G-protein-coupled receptors (GPCRs) are not uncommon and can lead to cardiovascular diseases. Given previously reported multiple factors associated with high mutation rates, we sorted the relative mutability of multiple human genes by (i) proximity to telomeres and/or (ii) high adenine and thymine (A+T) content. We extracted genomic information using the genome data viewer and examined the mutability of 118 ion channel and 143 GPCR genes based on their association with factors (i) and (ii). We then assessed these two factors with 31 genes encoding ion channels or GPCRs that are targeted by the United States Food and Drug Administration (FDA)-approved drugs. Out of the 118 ion channel genes studied, 80 met either factor (i) or (ii), resulting in a 68% match. In contrast, a 78% match was found for the 143 GPCR genes. We also found that the GPCR genes (*n* = 20) targeted by FDA-approved drugs have a relatively lower mutability than those genes encoding ion channels (*n* = 11), where targeted genes encoding GPCRs were shorter in length. The result of this study suggests that the use of matching rate analysis on factor-druggable genome is feasible to systematically compare the relative mutability of GPCRs and ion channels. The analysis on chromosomes by two factors identified a unique characteristic of GPCRs, which have a significant relationship between their nucleotide sizes and proximity to telomeres, unlike most genetic loci susceptible to human diseases.

## 1. Introduction

Ion channels are ubiquitous membrane proteins critical to almost all physiological processes, such as muscle contraction, neural transmission, and cardiac pace-maker function [1,2,3]. Ion channels are macromolecules with a relatively long size that are composed of subunits spanning the cell membrane. These channels are classified by the ion they allow the passage of—sodium (Na^+^), potassium (K^+^), calcium (Ca^2+^), or chloride (Cl^−^). Ion channels function as a gate via opening or closing by extracellular ligands [4], transmembrane voltage changes [5], or intracellular second messengers [6,7]. Mutations in ion channels are either causative or contributory to the pathogenesis of numerous disorders, such as cystic fibrosis [8,9], long-QT syndrome of the heart [10,11,12,13,14], heritable hypertension (e.g., Liddle’s syndrome) [15,16,17,18], hyperinsulinemia and hypoglycemia of infancy [19,20,21], hereditary nephrolithiasis (e.g., Dent’s disease) [22,23,24], and certain hereditary myopathies [25,26,27].

Ion channel proteins have been extensively investigated as drug targets, but it took much longer than expected for the outcomes of this research to be widely adopted clinically. Amlodipine besylate (brand name, Norvasc; Pfizer), which acts by blocking voltage-gated Ca^2+^ channels [28,29,30,31,32], was patented in 1982, and it has since become one of the bestselling anti-hypertensive medications. In 2019, amlodipine was the fifth most prescribed medication in the United States (US) [33]. As such, it has been perceived by drug developers that modulating ion channels is harder than targeting enzymes, kinases, and G-protein-coupled receptors (GPCRs). When designing a new drug, some would rather avoid interactions with ion channel proteins, particularly due to cardiovascular safety concerns. Although several attempts have been made to target ion channels, such as K^+^ channel blockers for autoimmune disease [34,35] and antiseizure medications [36,37,38,39,40], and antiarrhythmic treatments [41,42,43,44,45,46], targeting ion channels has resulted in a limited commercialization, as evidenced in the development of Ca^2+^ channel blockers for hypertension [47,48]. Nevertheless, a pathway for therapeutic success [49] and significant progress have been achieved with drugs that target Cl^−^ channels, such as cystic fibrosis transmembrane conductance regulator (CFTR) [50]. Moreover, Lidocaine, one of the most commonly prescribed medications in the US, is known to successfully target Na^+^ channels [51,52]. To a large extent, G-protein-coupled receptors (GPCRs) are vigorously studied as drug targets in the modern age [53], since they regulate diverse physiological processes by functioning as transmembrane transducers, which carry signals from extracellular ligands to effectors within the cell [54]. GPCRs can be linked to ion channels through their ligand, G protein, which is mediated by the direct physical interactions between G protein subunits and ion channels, including numerous voltage-dependent Ca^2+^ channels, as well as G-protein-activated K^+^ channels [55,56,57,58]. The majority of transmembrane signaling, which is accomplished by neurotransmitters and hormones, is largely dependent on the activation of G proteins by GPCRs [59]. In fact, nearly all druggable targets belong to one of the five primary families of proteins: GPCRs, ion channels, kinases, nuclear hormone receptors, or proteases [60]. Out of these five, GPCRs have been studied most intensively. In fact, nearly 30% of the global market share of therapeutic agents are associated with GPCR drugs [60,61]. GPCRs are the primary means by which cells can detect stimuli in the environment, as well as communicate with one another [62]. To this end, more than 60% of human hormones and roughly 30% of clinical drugs are able to activate approximately 350 GPCRs [62].

While the preference for GPCRs over ion channels as drug targets differs depending on the priority of manufacturers, the risk of targeting these proteins lies in unexpected mutations. Genetic mutation in the human genome is able to cause different responses to medications and is an increasing burden on public health. While 108 GPCRs are currently targeted by 34% (475) of Food and Drug Administration (FDA)-approved drugs, which account for more than 180 billion US dollars in annual sales globally, the likelihood and prevalence of mutations among drug-targeted GPCRs remain to be confirmed [61]. One previous report by the 1000 Genomes Project showed that an individual harbors an average of 68 missense mutations in the coding region of 33% of the GPCR drug targets [63]. Of these, eight mutations have been associated with clinically altered drug responses. Although ion channels are less preferred as drug targets in comparison to GPCRs, mutations of the host (human) gene leading to drug resistance have been previously reported in the North Indian population [64]. These data suggest a differential role of genetic polymorphisms of sodium voltage-gated channel alpha subunit 1 (SCN1A) and subunit 2 (SCN2A) in terms of both susceptibility to epilepsy, as well as drug response [64]. Three specific gene characteristics have been previously correlated with high rates of mutation in human chromosomes: (1) recombination rate [65], (2) proximity of a gene to its telomere, and (3) high adenine/thymine (A+T) content of a gene [66,67]. Of these three factors, we have shown previously that merely two of these factors—proximity to a telomere [68] and A+T content—are sufficient to predict the susceptibility of the genes to mutations and lead to monogenic and/or polygenic diseases [69,70]. To gain a better understanding through determination of the disease phenotype, the National Institute of Health (NIH) has released a list of 390 novel druggable genes under the title of ‘Commercializing Understudied Proteins from the Illuminating the Druggable Genome’ project (PA-19-034). Out of the 390 genes listed, 118 of the druggable candidates were classified as ion channels, while 143 of the genes were classified as GPCRs. Because genes encoding proteins that are more prone to mutations make less attractive drug targets, having a way to predict the mutability of these >260 ion channel and GPCR genes would help to prioritize which druggable targets to pursue for commercialization. 

In this report, we analyze how many of the genes that encode the 118 druggable ion channels and 143 GPCR genes identified by the NIH are able to match with one, both, or neither of the two previously identified predictive factors for genetic mutations: (i) proximity to telomeres and (ii) high A+T content. This predictive mechanism allows us to prioritize the 261 druggable proteins based on their relative mutability, as predicted by these aforementioned two factors. We then apply the same two factors to the 31 genes encoding ion channels and GPCRs targeted by currently FDA-approved drugs, providing the matching rate of these two factors with their respective drug targets, which include treatments for a wide range of human diseases in the cardiovascular, neurological, and other major organ systems. We demonstrate that this factor-druggable gene matching rate analysis is a useful tool to help systematically determine the relative mutability of these 261 novel druggable genes encoding ion channels and GPCRs, and thus inform the prioritization of drug development.

## 2. Materials and Methods

### 2.1. Database, Literature, and Open-Access Software

The publicly open NIH program announcement (PA-19-034) detailing the classification and identification of 390 understudied druggable genomes was utilized to acquire the list of 261 candidate genes encoding GPCRs and ion channels. The associated literature survey was performed until 9 December 2021, with emphasis on published title words with (1) ion channels since 2017 [4,5,9,10,11,12,13,14,16,26,27,30,33,39,40,41,42,44,46,47,48,53,54,59,61,69,70,71,72,73,74,75,76,77,78,79,80], (2) ion channel blockers since 2017 [41,81,82,83,84,85,86,87,88,89,90,91,92,93,94,95], (3) GPCR drugs since 2017 [53,60,61,96,97,98,99,100,101,102,103,104,105,106,107,108,109,110,111,112,113,114,115,116,117,118,119,120,121,122,123,124,125,126,127,128,129,130,131,132,133], (4) hypertension and GPCR [134,135,136,137,138,139,140,141,142,143], (5) AD and GPCR [144,145,146,147,148], and (6) Down syndrome [149,150,151,152,153,154,155,156,157,158,159,160,161,162,163,164,165,166,167,168,169,170,171,172,173,174,175,176,177,178,179,180,181,182,183]. This literature survey was systematically conducted following the method that has been used previously [69].

In order to measure the distance between a designated gene of interest and its telomere, we utilized the NIH Genome Data Viewer (https://www.ncbi.nlm.nih.gov/genome/gdv/) accessed on 1 February 2022. To calculate the A+T content present in a designated gene of interest, a GC content calculator (https://www.biologicscorp.com/tools/GCContent/#.XvctCi-z2uV) accessed on 1 February 2022 was used. This resulted in the acquisition of the compositions of adenine and thymine along with the full-length sizes of the nucleotide [69,70] for each gene.

### 2.2. Approximation of Proximity to a Telomere

We have followed a previously established method in approximating the proximity of a gene to its telomere [70]. Therefore, in this study, we have determined the position of each gene encoding an ion channel or GPCR and its distal end locus of each arm (telomere) according to the following principle:

If the recombination frequency is less than (≤) 50 centimorgan (cM), genes are linked and are therefore proximal to one another: (1) If the recombination frequency is higher than 50 cM, genes are not linked and are therefore distal to one another: (2) (where 1 cM ≅ 1 million base pair (Mbp) [184]).

### 2.3. Data Plot and Statistical Methods

Relevant heatmap plots, bar graphs, as well as box and violin plots of the data obtained during analysis with the genome data viewer were generated via the use of Prism. Similarly, all statistical analyses were performed using Prism (version 9.3.0, GraphPad Software Inc., San Diego, CA, USA) as well. A Shapiro–Wilk normality test (α < 0.05) was used to confirm that the data were normally distributed. Unless stated differently, all comparisons of two groups at a time were conducted through the use of two-sided unpaired t-tests. In order to compare more than two groups at a time, after conducting one-way analysis of variance (ANOVA), Tukey’s multiple comparisons test was used. For all statistical analyses, differences were considered significant at *p* < 0.05. *p*-values are denoted in the figures/legends as * *p* < 0.05, ** *p* < 0.01, and *** *p* < 0.005.

## 3. Results

### 3.1. Outlines of Two Factors in Genes Encoding 261 Novel Druggable Genomes

**Figure 1:**
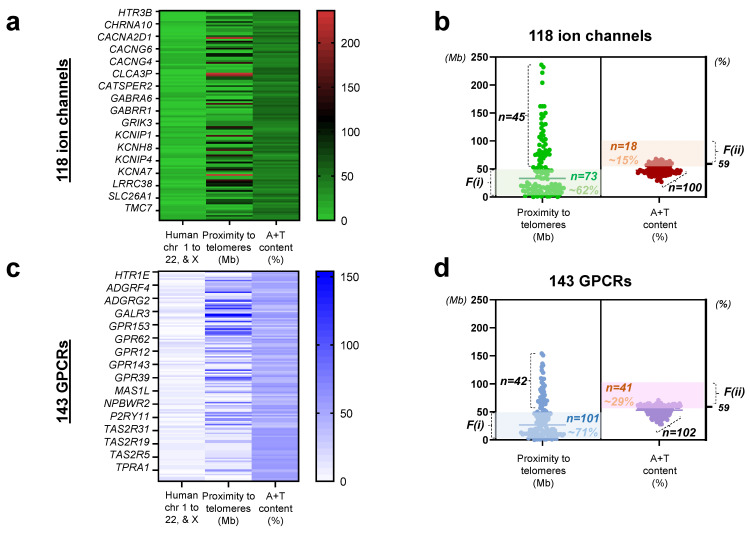
Relative mutability of genes encoding ion channels and GPCRs (**a**) a color-coded map illustrating distributions of 118 genes encoding ion channels with respect to the human chromosome 1 through 22 and X (color code range 1 to 23), proximity to telomeres, and A+T content (range 0 to 100%), respectively. Note that only select genes (not all 118) are marked on the left y axis. The reference proximity to telomeres (range 0 to 250 Mb) with coded colors is shown on the right. (**b**) Scatter plots displaying relative mutability of druggable ion channels by proximity to telomeres (left) and A+T content (**right**). Shaded regions with light green (**left**) and light brown (right) demonstrate genes satisfying either of the two factors. Genes outside the shaded regions (*n* = 45 by F(i); *n* = 100 by F(ii)); relatively less mutable. (**c**) the color-coded map illustrating distributions of 143 genes encoding GPCRs with respect to the human chromosome 1 through 22 and X (color code range 1 to 23), proximity to telomeres (range 0 to 150), and A+T content (range 0 to 100), respectively. Note that select genes (not all 143) are marked on the left y axis. The reference number with coded color is shown on the right, (**d**) scatter plots exhibiting relative mutability of druggable GPCRs by proximity to telomeres (**left**) and A+T content (**right**). Shaded regions with light blue (**left**) and light purple (**right**) show genes satisfying either of the two factors. Genes outside the shades (*n* = 42 by F(i); *n* = 102 by F(ii)) are relatively less mutable.

We sought to examine the relative mutability of the druggable ion channel and GPCR genes belonging to the 361 understudied druggable genomes identified by the National Institute of Health (NIH PA-19-034). The heatmap plots of the 118 ion channels and 143 GPCRs were created to demonstrate (1) relative distribution of the genes across all the chromosomes (1 to 23, in which 23 corresponds to the chromosome X), (2) the distance of a human gene from its telomere in million bases (Mb; 0 to 200), and (3) A+T composition of the gene (%). Specifically, the intensity of the overall tone in color is relatively weak in the chromosomal distribution of the 118 ion channels from chromosome 1 to 22, and X (assigned as 23; Figure 1a, the left panel) being comparatively strong in the distribution of proximity to telomeres (Figure 1a, the middle panel), and at the intermediate range in A+T content (Figure 1a, the right panel) (Tables S1 and S2). 

As these data are organized in scatter plots, factor (i) or the location of the genes is noticeably skewed to being closer to their telomeres while still displaying a wide range, whereas factor (ii) forms a distinct cluster near 50% (Figure 1b). Roughly 62% of the 118 genes encoding ion channels satisfied factor (i) located < 50 Mb from the telomere, whereas only ~15% of the 118 ion channel genes met factor (ii) with an A+T content greater than 59%, which is the human genome-wide average [67]. For the 143 genes encoding GPCRs, the range of distances to telomeres was narrower (0 to 150 Mb; Figure 1c) than for ion channels (up to 250 Mb). The number of genes encoding druggable GPCRs that met F(i) (gene proximity < 50 Mb from its telomeres) was greater than for ion channels at ~71% vs. 62%. Consistent with the relative mutability estimated by proximity to telomeres, ~29% of the 143 genes encoding GPCRs satisfied F(ii) at >59% (Figure 1d), whereas a smaller portion or ~15% met F(ii) for ion channels.

### 3.2. Prioritization of Druggable Ion Channels per the Relative Mutability

**Figure 2:**
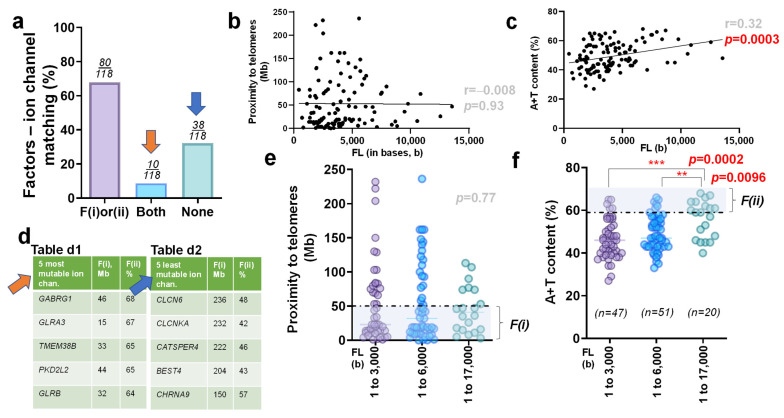
Relative mutability of druggable ion channels by two factors (**a**) Bar graph, which demonstrates the matching rate of genes encoding 118 druggable ion channels with both telomere proximity, F(i), and nucleotide composition, F(ii). Genes are sorted into three separate groups: those that satisfy one of the two factors, (F(i) or (ii)), those meeting both F(i) and F(ii) (Both, denoted with orange arrow), and those satisfying neither F(i) nor F(ii) (None, denoted with blue arrow) (**b**) Scatter plot showcasing the correlation between the full-length size of genes that encode ion channels and their proximity to telomeres. The Pearson correlation coefficient for this relationship is r = −0.008, with a level of statistical significance of *p* = 0.93. (**c**) Scatter plot showcasing the correlation between the full length of genes that encode ion channels and their A+T content. The Pearson correlation coefficient for this relationship is r = 0.32, with a level of statistical significance of *p* = 0.0003. (**d**) The five primary candidates (*GABRG1, GLRA3, TMEM38B, PKD2L2,* and *GLRB*) that have been sorted as the genes with relatively high mutability (the ‘Both’ group; Table d1). Five alternative candidate genes (*CLCN6, CLCNKA, CATSPER4, BEST4*, and *CHRNA9*), which have been sorted as the genes with relatively low mutability (the ‘None’ group; Table d2). (**e**) Scatter plot showcasing the proximity to telomeres of genes encoding 118 ion channels with respect to three sub-groups, which were determined by the full-length size of the gene (unit: base or b). The dotted horizontal line demarcates 50 Mb. After one-way ANOVA, no significant differences (*p* = 0.77) were found. (**f**) Scatter plot demonstrating the A+T content of genes encoding 118 ion channels with respect to the full-length of each gene. The dotted horizontal line demarcates 59%. Significant statistical differences were determined between the shortest (1 to 3000 bp) and longest size group (6001 to 17,000 bp) at *p* = 0.0002, as well as the intermediate (3001 to 6000 b) vs. the longest size group (6001 to 17,000 b) at *p* = 0.0096, respectively. **, *p* < 0.01; ***, *p* < 0.005.

Next, we were able to identify the matching rate between druggable ion channel genes and the two aforementioned factors. We determined that roughly 68% of the ion channel genes were either sufficiently proximal to telomeres or contained high A+T content (*n* = 80/118). On the other hand, approximately 32% of the genes (38 of 118) encoding druggable ion channels satisfied neither factor. Moreover, fewer than 9% of the genes (10 of 118) met the conditions of both factors. As in the previous reports [69,70], the groups of ’Both (meeting both factors)’ and ‘None (meeting neither factor)’ were particularly noteworthy, since they indicated that a gene was a relatively more or less mutable target, respectively, thereby potentially informing the commercialization potential of drugs targeting ion channels associated with these genes (Figure 2a).

When examining the correlation between the molecular size of a particular gene and each of the two factors, the Pearson coefficient (r = −0.008) indicated no significant correlation (*p* = 0.93) between the full-length sizes of the genes and the proximity of each gene to its nearest telomere in druggable ion channels (Figure 2b). However, as is consistent with the prior report [70], there was a significant correlation between the full-length size of a gene and its A+T content (r = 0.32; *p* = 0.0003) (Figure 2c).

Next, we examined each of the specific genes that matched with ‘both’ or ‘none’ of the two factors, then prioritized the top five ion channels based on their relative mutability. This allowed us to produce the names of five candidate genes (*GABRG1, GLRA3, TMEM38B, PKD2L2,* and *GLRB*), which showed relatively higher mutability corresponding to the ‘Both’ group (orange arrow). Alternatively, five other candidate genes (*CLCN6, CLCNKA, CATSPER4, BEST4*, and *CHRNA9*) were sorted as the ion channel genes with relatively lower mutability corresponding to the ‘None’ group (blue arrow; Figure 2d).

We then analyzed each gene alongside F(i) and F(ii) with respect to the length of each nucleotide. Following previous analyses [69,70], we grouped all analyzed genes into three categories of length: 1–3000 bases (*n* = 47), 3001–6000 bases (*n* = 51), and 6001–17,000 bases (*n* = 20). Through statistical analysis, we determined that there was no significant difference in the proximity of a gene to its telomere with respect to the gene’s full-length size (*p* = 0.77). However, after conducting a one-way analysis of variance (ANOVA) and pair-wise comparisons using post hoc Tukey’s test, it became clear that there was a significant difference (*p* = 0.0002) in the A+T content of a gene with respect to the full-length size (bases) of the gene between the longest and shortest sub-groups. Furthermore, we also determined that there was a significant difference (*p* = 0.0096) between the intermediate and longest genes as well (Figure 2e,f).

Our discovery of this subset of ion channels with a relatively low predicted mutability rate implies that commercialized ion channel drugs could fit into any of these three categories meeting (1) one of the two factors or (2) both or (3) none.

### 3.3. Prioritization of Druggable GPCRs per the Relative Mutability

**Figure 3:**
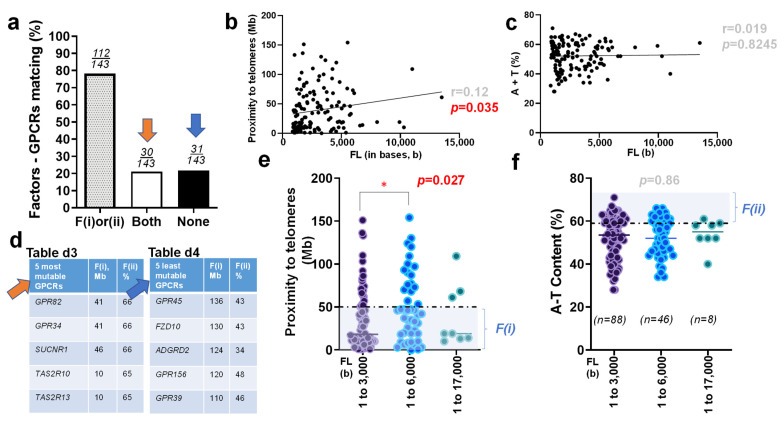
Relative mutability of druggable GPCRs by two factors (**a**) Bar graph, which summarizes the matching rate of genes that encode 143 druggable GPCRs with their F(i), proximity to telomeres and F(ii), their A+T content. Genes are divided into three groups: those that meet one of the two factors, (F(i) or (ii)), those that satisfy both F(i) and F(ii) (Both, denoted with orange arrow), and those that meet neither F(i) nor F(ii) (None, denoted with blue arrow) (**b**) Scatter plot that exhibits the relationship between the full-length size of genes that encode GPCRs and their associated proximity to telomeres. Statistical analysis resulted in a Pearson correlation coefficient of r = 0.12, with a level of statistical significance of *p* = 0.035. (**c**) Scatter plot that displays the relationship between the full length of genes that encode GPCRs and their A+T content. The Pearson correlation coefficient was determined to be r = 0.019, while the level of statistical significance was *p* = 0.8245. (**d**) Five primary candidates (*GPR82, GPR34, SUCNR1, TAS2R10*, and *TAS2R13*) that have been sorted as genes with relatively high mutability (the ‘Both’ group; Table d3). Moreover, five other candidate genes (*GPR45, FZD10, ADGRD2, GPR156*, and *GPR39*) have been sorted as genes with relatively low mutability (the ‘None’ group; Table d4). (**e**) Scatter plot showcasing the proximity to telomeres of genes encoding 143 GPCRs, which have been divided into three sub-groups by the full-length size of their gene (bp). A dotted horizontal line demarcates 50 Mb. A statistically significant difference between the smallest (1 to 3000 bp) and intermediate size groups (3001 to 6000 b) was determined as *p* = 0.027 (**f**) Scatter plot indicating the relationship between the A+T content of genes encoding 143 GPCRs with respect to the full-length of each of the genes. A dotted horizontal line demarcates 59%. No significant difference was found among any of these groups. * *p* < 0.05.

Moreover, we also compared the matching rate between these two aforementioned factors and 143 druggable GPCRs (Table S3). Of these GPCRs, 78% of 143 genes met either proximity to telomeres or high A+T content (*n* = 112/143). Notably, more than 20% of the genes (30 of 143) that encode GPCRs satisfied both F(i) and F(ii). On the other hand, 22% of these genes (31 of 143) met neither factor (Figure 3a). To examine the correlation between molecular size and each of the factors, the Pearson coefficient (r = 0.12) was determined. This analysis indicated the existence of a significant correlation (*p* = 0.035) between the full-length sizes of the 143 genes encoding druggable GPCRs and their proximity to telomeres. Unlike previous reports, this significant correlation between molecular size and telomere proximity has never been detected in any genomic analysis [69,70]. However, no significant difference between the full-length size of the GPCR-encoding genes and their associated A+T content was detected (r = 0.019; *p* = 0.8245) (Figure 3b,c).

Next, we assessed each of the specific genes that matched with ‘both’ or ‘none’ of the two factors and prioritized the top five GPCRs of each category based on their relative mutabilities. This strategy enabled us to sort out five candidate genes (*GPR82, GPR34, SUCNR1, TAS2R10*, and *TAS2R13*), which showed a relatively higher mutability, corresponding to the ‘Both’ group (denoted with orange arrow). Conversely, five other candidate genes (*GPR45, FZD10, ADGRD2, GPR156*, and *GPR39*) were selected as the GPCR genes with a relatively lower mutability, which corresponded to the ‘None’ group (denoted with blue arrow; Figure 3d).

We then organized both factors with respect to the length of individual nucleotides. Per the previous analysis [69,70], we grouped all of the examined genes into three categories: 1–3000 bases (*n* = 88), 3001–6000 bases (*n* = 46), and 6001–17,000 bases (*n* = 8), respectively. Statistical analysis conducted on these groups suggested that there was a significant difference in the proximity of a gene to its telomere between the shortest and intermediate size genes (*p* = 0.027). After a one-way ANOVA, pair-wise comparisons using post hoc Tukey’s test revealed no significant difference in the A+T content of a gene with respect to its full-length size (bases) (Figure 3e,f).

### 3.4. Mutability of Ion Channels and GPCRs Targeted by the Commercialized Drugs

To glean a more comprehensive understanding of the genomic characteristics that are associated with mutations in novel druggable genomes in terms of known commercialized drugs, we examined the mechanisms of action of the drugs on the market, which target either ion channels or GPCRs. Primarily, the portion of the survey on commercialized drugs targeting ion channels resulted in eleven pharmacological agents, from those targeting L-type CaV (amlodipine) to those targeting *TRAAK-1* (Riluzole) (Table 1). With the exception of 2 drugs, 9 out of 11 drugs targeting ion channels satisfied either F(i) or F(ii). Specifically, for amlodipine, cana1c demonstrated a sufficient proximity to its telomere at 1.9 Mb. For pregabalin, *CACNA2D1* satisfied F(ii) or high A+T content at 64%. For sotalol, *KCNH2* satisfied proximity to its telomere at 8 Mb. For flecainide, *SCN5A* met F(i) at 38 Mb. For ziconotide, *CACNA1B* met proximity to its telomere at 0.1 Mb. For varenicline, *CHRNA4* satisfied proximity to its telomere at 1 Mb. Similarly, for retigabine, *KCNQ2* met F(i) at 1 Mb. For VU0456810, *GIRK2* met both proximity to its telomere at 9 Mb and high A+T content at 60%. In the case of riluzole, *KCNK4* met neither proximity to its telomere nor high A+T content. Similarly, for diazepam, *GABRB3* satisfied neither F(i) nor F(ii). Among these 11 drugs targeted by ion channels, 82% (9 out of 11) satisfied either F(i) or F(ii) (Table 1).

The measurements of distances to telomeres and nucleotide compositions on genes encoding GPCRs targeted by the commercialized drugs demonstrated that fifteen different genes—encoding *PTGFR, OPRD1, HTR1A, GLP1R, PTGIR, SMO, PTH1R, P2RY12, NR1L2, ADRB2, GLP1R, MTNR1A, OPRM1, ADRB1*, and *HTR1A*—satisfied either proximity to telomeres at < 50 Mb or high A+T content at > 59%. On the other hand, five additional genes encoding GPCRs, *S1PR1, TACR1, HCRT2, CASR*, and *DRD3*, met neither of the two factors (Table 2).

### 3.5. Thirty-One Genes Encoding Ion Channels and GPCRs Targeted by Approved Drugs

**Figure 4:**
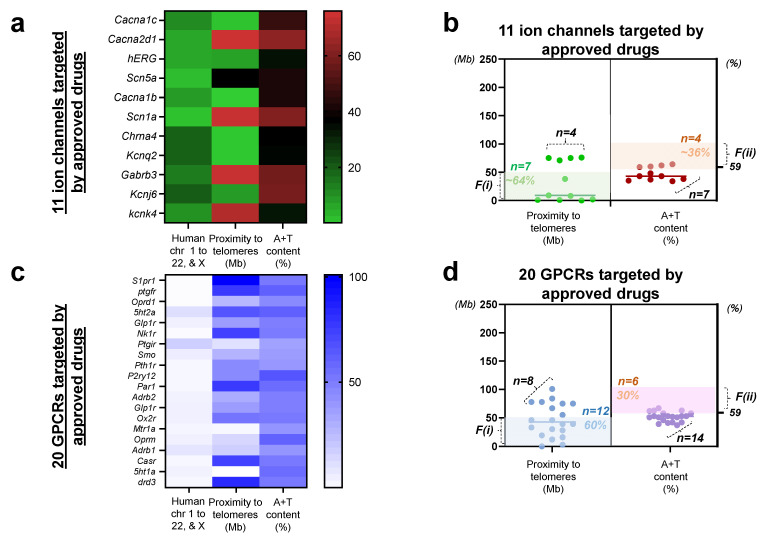
Relative mutability of ion channels and GPCRs targeted by approved drugs (**a**) the color-coded map illustrating distributions of 11 genes encoding ion channels targeted by commercialized drugs with respect to the human chromosome 1 through 22 and X (color code range 1 to 23), proximity to telomeres (range 0 to 100), and A+T content (range 0 to 100), respectively. Note that all 11 genes are marked on the left (y) axis. The reference number with coded color is shown on the right (**b**) scatter plots displaying relative mutability of 11 ion channels by proximity to telomeres (**left**) and A+T content (**right**). Shaded regions with light green (**left**) and light brown (**right**) demonstrate genes satisfying either of the two factors. Note that genes outside the shaded regions (*n* = 4 by F(i); *n* = 7 by F(ii)) are relatively less mutable. (**c**) the color-coded map illustrating distributions of 20 genes encoding GPCRs with respect to the human chromosome 1 through 22 and X (color code range 1 to 23), proximity to telomeres (range 0 to 100), and A+T content (range 0 to 100), respectively. Note that all 20 genes are marked on the left (y) axis. The reference number with coded color is shown on the right (**d**) scatter plots exhibiting relative mutability of 20 GPCRs by proximity to telomeres (**left**) and A+T content (**right**). Shaded regions with light blue (**left**) and light brown (**right**) show genes satisfying either of the two factors. Note that genes outside the shaded regions (*n* = 7 by F(i); *n* = 15 by F(ii)) are relatively less mutable.

**Figure 5:**
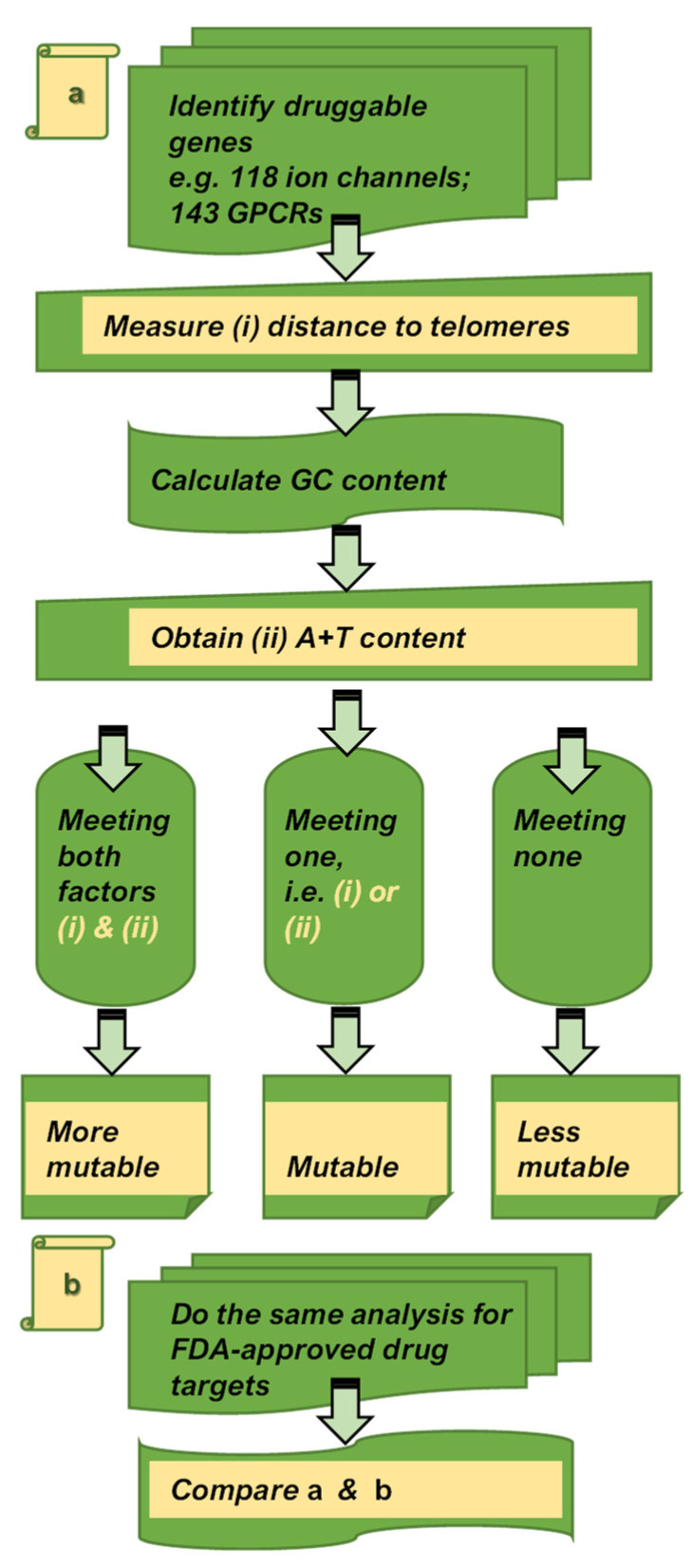
Flow chart summarizing how chromosomal analyses are conducted in this study (**a**) diagrams showing the logical flow of tasks on druggable genomes (**b**) diagrams displaying tasks on FDA-approved drug targets and amalgamation of the dataset in a and b.

We next assessed the two factor characteristics of genes encoding ion channels and GPCRs targeted by 31 commercialized drugs. The heatmap plots of 11 genes encoding ion channels demonstrate a similar spectrum to the 118 druggable ion channels (Figure 1), as assessed on the range of values in chromosome number (1 to 23, in which 23 corresponds to the chromosome X), proximity to telomeres (Mb; 0 to 100), and A+T content (%; between 35 and 70). Specifically, the intensity of the overall tone in color is relatively weak in the chromosomal distribution of 11 ion channels from chromosome 1 to 22, and X (assigned as 23; Figure 4a, the left panel), strong in the distribution of proximity to telomeres (Figure 4a, the middle panel), and at the intermediate range in A+T content (Figure 4a, the right panel). As these data are organized in scatter plots, F(i) shows a widespread distribution over 0 to 100 Mb in proximity to telomeres, while F(ii) forms a narrower distribution near 50% (Figure 4b). Approximately 64% of 11 genes encoding ion channels satisfied F(i) at <50 Mb, while ~36% of 11 ion channel genes met F(ii) at >59%. A similar spectrum in color is found in proximity to telomeres of 20 genes encoding GPCRs (0 to 100 Mb; Figure 4c). As compared to ion channels targeted by approved drugs (Figure 4a), a greater number of genes encoding GPCRs satisfied F(i) at 60% at <50 Mb, while 30% of 20 genes encoding GPCRs met F(ii) at >59% (Figure 4d). To this end, we provide the logical flow of work conducted in the present analyses (Figure 5).

## 4. Discussion

To better understand the mutability of druggable genomes, we applied two factors associated with high mutation rates to the genes encoding ion channels and GPCRs. The novelty of this work lies in our finding that the two factors identify a unique feature of GPCRs, having a significant relationship between their nucleotide sizes and proximity to telomeres unlike the majority of genetic loci susceptible to human diseases [69,70]. In the human genome, it has been reported that more than 400 genes encoding ion channels mediate ion fluxes across cell membranes [190]. A significantly smaller percentage (~15%) of currently used drugs target ion channels [49]. In contrast, more than 800 members of GPCRs are known so far, forming the largest family of cell-surface receptors [120]. As expected from this difference in numbers (400 ion channels vs. 800 GPCRs), 34% of all drugs approved by the US FDA act on over 100 GPCRs, and clinical trials have explored >300 new GPCR agents [120].

Using the outlines of druggable genomes in color-coded maps (Figure 1a vs. Figure 1c), we first noticed that ion channels are bigger in size than GPCRs by the upper limit (250 vs. 150 Mb). Given the previous reports [69,70], this suggested to us that druggable ion channels are less likely to meet the factor (i) or proximity to telomeres than druggable GPCRs are. As displayed in Figure 1b,d, we found that relatively small portions of ion channels (62%) and GPCRs (71%) met the factor (i) when compared with the high matching rates reported in >100 genes at ~80 to 91% [69,70]. Twice as many GPCRs (29%) met the factor (ii) or high A+T content than ion channels did (15%). This also suggested that the relationship or correlation between the molecular size and A+T content in ion channels differed from that of GPCRs. 

The matching rate of either of the two factors with druggable ion channels (Figure 2a) was comparable to those analyzed in genetic mutant loci of congenital heart disease (CHD) and thoracic aortic aneurysm (TAA), which showed a matching rate at ~74% (62 of 84 genes, collectively) [69]. Consistent with the previous reports [69,70], there was a typical non-significant relationship in full-length (FL) size of the druggable ion channels with respect to the proximity to telomeres. A typical feature of statistical significance in FL–A+T content was also noted in druggable ion channels (Figure 2b,c). From the short lists of more vs. less mutable genes, *TMEM38B* draws our attention, as it belongs to one of the five most mutable ion channels due primarily to high A+T content (Figure 2d). When 118 druggable ion channel genes were divided into three groups per their molecular size, a typical correlation between FL–proximity, as well as FL–A+T content, was revealed (Figure 2e,f), which is consistent with the result obtained from 108 genes causing congenital disorder of the brain where *TMEM67* mutants were reported [70,74,77]. Overall, 118 genes encoding druggable ion channels were relatively less mutable and longer in molecular size than 143 genes encoding druggable GPCRs (Figure 2a vs. Figure 3a).

Next, the matching rate of one of the two factors with druggable GPCRs (Figure 3a) was reminiscent of the result obtained from Alzheimer’s disease (AD) and monogenic hypertension (MH), which resulted in the matches of either of the two factors and the disease at ~84% (59 of 70 genes susceptible to AD; 66 of 79 genes causative of MH) [69,70]. Indeed, GPCRs become important targets in understanding the molecular mechanisms underlying AD [144,145,146,147,148] and hypertension [134,135,136,137,138,139,140,141,142,143]. Inconsistent with the prior reports [69,70], however, there was an atypical significant relationship in proximity to telomeres with respect to the molecular size (FL) of the druggable GPCRs (Figure 3b,c). Given the human genome-wide average at 59% [67], most mutable GPCRs sorted in this study have demonstrated their A+T content at unusually high compositions of 65–66% (Figure 3d). When 143 druggable GPCRs were divided into three groups per their molecular size, the atypical correlation between FL–proximity was detected (Figure 3e in comparison with Figure 3f), which is a unique feature of the GPCRs that has never been found in any of the human genes associated with monogenic or polygenic disease analyzed previously [69,70].

As we further analyzed the approved drugs targeting ion channels and GPCRs, it became clear that ion channels with much smaller molecular sizes than the 118 listed druggable genomes are commercialized into drugs, as inferred from their proximity ranges being located between 0 to less than 100 Mb (Figure 4a compared to Figure 1a). As assessed by the molecular sizes of ion channels targeted by approved drugs (Table 1), the observation that drugs are targeting molecules with the nucleotide sizes of less than 10,000 bp is confirmed with three exceptions: (1) amlodipine targeting *CACNA1C* with 13,744 bp, (2) lacosamide targeting *SCN1A* showing 13,079 bp, and (3) VV0456810 targeting *KCNJ6* with 19,657 bp. Our data (Figure 1 and Figure 4) indicate that if one aims at designing new drugs, one will follow the trend revealed in these analyses:

- Figure 1: There are >260 candidates proposed (ion channels and GPCRs), but sizes are wider in range.
- Figure 4: In reality, drug manufacturers target genes with small(er) sizes in ion channels and GPCRs.

Why? If the target is long in size, can more side effects arise? Specificity will decrease because the longer the sequences, there is a higher chance of (1) having multiple same/similar fraction of sequences and (2) having higher A+T content.

Even though the smaller-size molecules were chosen to be commercialized into drugs (Figure 4b), the matching rates of proximity to telomeres and ion channels are similar between the druggable and the drug-targeted ion channel genes (62% vs. 64%). As far as mutability is concerned, however, much higher matching rate of ion channels and A+T content that we found from drug-targeted genes (36%) as compared to those of druggable ion channels (15%) may be due to the relatively longer molecular sizes of ion channels (Figure 1b vs. Figure 4b) as compared to those of GPCRs (Figure 1d vs. Figure 4d). 

At a 10% margin for the factor (i), the matching rates of either of the two factors and the gene under investigation are similar between the druggable GPCRs (71% and 29%; Figure 1d) and the GPCRs targeted by approved drugs (60% and 30%; Figure 4d). Collectively, these two factors organized in specific numbers support the idea that the same proportions (2 of 11 genes vs. 5 of 20 genes) of the molecules show the least mutability in 11 ion channels and 20 GPCRs targeted by approved drugs (marked in grey in Table 1 and Table 2).

Three genomic factors of recombination rate, proximity to telomeres, and high A+T content [67], in addition to a defect in cell division or chromosome mis-segregation, resulting in aneuploidy [149,158,163,164,165,169,176], were reported to be associated with high mutation rates [67]. Although the utility of matching rate analysis has been demonstrated with the highest predictability at ~91% in 108 genes [69,70], there is a limitation in the applicability of matching rate analysis to birth defects, such as Down syndrome, which is caused by trisomy 21 [149,158,163,164,165,169,176]. 

## 5. Conclusions

Among the 118 examined druggable ion channels, 80 of the human genes that encode ion channels were suitably proximal to their telomeres at <50 Mb or contained high A+T content at >59%, suggesting that 68% of these genes are mutable. On the other hand, 38 of 118 genes that encode druggable ion channels were determined to be relatively less mutable, since they satisfied neither of the two factors. Of the 143 genes encoding druggable GPCRs, 112 of these genes were able to meet either F(i) or F(ii), suggesting that 78% of these GPCR genes have high mutability. Conversely, 31 of the 143 genes encoding druggable GPCRs were determined to be relatively less mutable, thereby meeting neither of the two aforementioned factors. Moreover, eleven ion channels and twenty GPCRs targeted by FDA-approved drugs were suggestive that ion channels have relatively longer molecular sizes than GPCRs, resulting in a higher likelihood of meeting F(ii). Overall, 118 genes encoding druggable ion channels were relatively less mutable and longer in molecular sizes than 143 genes encoding druggable GPCRs. Compared to the approved drugs targeting ion channels and GPCRs, however, ion channels with much smaller molecular sizes than 118 druggable ion channels are targeted on the market and commercialized as drugs. Similarly, relatively smaller-size GPCRs at the range of less than 10,000 bp are chosen to be materialized into GPCR drugs. Both cases of intended selections in shorter-size molecules support the previous findings [69,70] that a shorter nucleotide length of a gene under investigation corresponds to a reduced likelihood to satisfy the factor (ii) or high A+T content, leading to drug targets with relatively less mutability.

Investigators in the field use laboratory animals aiming to test their hypotheses ultimately for translational purposes. It is not uncommon to find that a pharmacological approach that worked in rodents fails to reproduce the expected outcome when tested in human clinical trials. This study provides a guideline of prioritizing a more reliable drug target by relative mutability based on two factors using the genomes of humans. With all conditions, such as safety, toxicity, and efficacy of drugs, comparable, and if there are two or more such drug targets, a less mutable pharmacological target can be determined, augmenting decision making on which drug target will result in a consistent outcome in laboratory animals and humans. This means that the same analysis presented in this study can be applied to diverse animal genomes available at the National Center for Biotechnology Information database.

## Figures and Tables

**Table 1:** Two factor characteristics of 11 ion channels targeted by approved drugs.

Drug	Target Gene	Chr	Gene Loci	Telomere Loci	Proximity (Mb)	A,T (%)	A+T (%)	FL Size (bp)	References
Amlodipine	*CACNA1C*	12	1.9	0	1.9	23,25	48	13,744	[185]
Pregabalin	*CACNA2D1*	7	82	158	76	31,33	64	7542	[185]
Sotalol	*HERG (KCNH2)*	7	150	158	8	16,19	35	4292	[185]
Flecainide	*SCN5A*	3	38	0	38	21,22	43	8516	[185]
Ziconotide	*CACNA1B*	9	138	138.1	0.1	21,22	43	9792	[185]
Lacosamide	*SCN1A*	2	166	241	75	30,32	62	13,079	[185]
Varenicline	*CHRNA4*	20	63	64	1	17,21	38	5583	[185]
Retigabine	*KCNQ2*	20	63	64	1	17,20	37	9163	[185]
Diazepam	*GABRB3*	15	26	101	75	28,31	59	5767	[185]
VU0456810	*KCNJ6*	21	37	46	9	29,31	60	19,659	[185,186,187]
Riluzole	*KCNK4*	11	64	135	71	14,20	34	1829	[188]

*** marked in grey indicating the relatively less mutable characteristics, meeting none of the two factors.

**Table 2:** Two factor characteristics of 20 GPCRs targeted by approved drugs.

Drug	Target Gene	Chr	Gene Loci	Telomere Loci	Proximity (Mb)	A,T (%)	A+T (%)	FL Size (bp)	References
Siponimod	*S1PR11*	11	101	0	101	24,29	53	2778	[189]
Latanoprostene bunod	*PTGFR*	1	78	0	78	30,33	63	5429	[189]
Hycodan	*OPRD* or *OPRD1*	1	28	0	28	21,25	46	9317	[60]
Rexulti	*5HT2A* or HTR2A	13	46	113	67	31,32	63	5415	[60]
Trulicity	*GLP1R*	6	39	0	39	24,27	51	6682	[60]
Varubi	*NK1R* or *TACR1*	2	75	0	75	25,27	52	4779	[60]
Uptravi	*PI2R* or *PTGIR*	19	46	58	12	17,20	37	2078	[60]
Odomzo	*SMO*	7	129	159	30	17,22	39	3977	[60]
Tymlos	*PTHR1* or *PTH1R*	3	46	0	46	20,21	41	2153	[60]
Kengreal	*P2Y12* or *P2RY12*	3	151	197	46	34,33	67	2244	[60]
Zontivity	*PAR1* or *NR1I2*	3	197	197	0.1	33,25	58	2232	[60]
Striverdi Respimat	*ADRB2*	5	148	181	33	23,28	51	2013	[60]
Adlyxin	*GLP1R*	6	39	0	39	24,27	51	6682	[60]
Belsomra	*OX2R* or *HCRTR2*	6	55	0	55	24,29	53	1952	[60]
Hetlioz	*MTR1A* or *MTNR1A*	4	186	189	3	19,23	42	1289	[60]
Symproic	*OPRM* or *OPRM1*	6	154	170	16	30,32	62	15,143	[60]
Northera	*ADRB1-3* or *ADRB1*	10	114	133	19	18,24	42	3039	[60]
Parsabiv	*CASR*	3	122	197	75	26,27	53	10,062	[60]
Aristada	*5HT1A* or *HTR1A*	5	197	197	0.1	33,25	58	2232	[60]
Vraylar	*DRD3*	3	114	198	84	25,28	53	2770	[60]

*** marked in grey indicating the relatively less mutable characteristics, meeting none of the two factors.

## Data Availability

The data presented in this study are available in Supplementary Material as Tables S1 and S2.

## Supplementary Materials

The following supporting information can be downloaded at: https://www.mdpi.com/article/10.3390/biomedicines10030594/s1, Table S1: Two factor characteristics of 118 druggable ion channels; Table s2: Identification of 118 druggable ion channel types; Table S3: Two factor characteristics of 143 druggable GPCRs.

## References

[1] Alothaid H., Aldughaim M.S.K., El Bakkouri K., Al Mashhadi S., Al-Qahtani A.A. (2020). Similarities between the effect of SARS-CoV-2 and HCV on the cellular level, and the possible role of ion channels in COVID-19 progression: A review of potential targets for diagnosis and treatment. Channels.

[2] Bertagna F., Lewis R., Silva S.R.P., McFadden J., Jeevaratnam K. (2021). Effects of electromagnetic fields on neuronal ion channels: A systematic review. Ann. N. Y. Acad. Sci..

[3] Joukar S. (2021). A comparative review on heart ion channels, action potentials and electrocardiogram in rodents and human: Extrapolation of experimental insights to clinic. Lab. Anim. Res..

[4] Sun X., Zhou R., Lei Y., Hu J., Li X. (2020). The ligand-gated ion channel P2X7 receptor mediates NLRP3/caspase-1-mediated pyroptosis in cerebral cortical neurons of juvenile rats with sepsis. Brain Res..

[5] Alvarado D., Cardoso-Arenas S., Corrales-Garcia L.L., Clement H., Arenas I., Montero-Dominguez P.A., Olamendi-Portugal T., Zamudio F., Csoti A., Borrego J. (2020). A Novel Insecticidal Spider Peptide that Affects the Mammalian Voltage-Gated Ion Channel hKv1.5. Front. Pharmacol..

[6] Ackerman M.J., Clapham D.E. (1997). Ion channels--basic science and clinical disease. N. Engl. J. Med..

[7] Munemasa S., Oda K., Watanabe-Sugimoto M., Nakamura Y., Shimoishi Y., Murata Y. (2007). The coronatine-insensitive 1 mutation reveals the hormonal signaling interaction between abscisic acid and methyl jasmonate in Arabidopsis guard cells. Specific impairment of ion channel activation and second messenger production. Plant Physiol..

[8] Davis P.B., Drumm M., Konstan M.W. (1996). Cystic fibrosis. Am. J. Respir. Crit. Care Med..

[9] Konstan M.W., Pasta D.J., VanDevanter D.R., Wagener J.S., Morgan W.J., Scientific Advisory G., Scientific Advisory Group, The Investigators and Coordinators of ESCF (2021). Epidemiologic Study of Cystic Fibrosis: 25 years of observational research. Pediatr. Pulmonol..

[10] Chen J., Song Y., Gu J., Chen L. (2021). Reprogramming of a human induced pluripotent stem cell line from a long QT syndrome patient harboring a heterozygous mutation of c.1537C>T in SCN5A gene. Stem Cell Res..

[11] Garcia Gozalo M., Bermejo Arnedo I., de Vera McMullan P. (2021). KCNQ1 gene mutation and epilepsy in patient with long QT syndrome. Med. Clin..

[12] Gessner G., Runge S., Koenen M., Heinemann S.H., Koenen M., Haas J., Meder B., Thomas D., Katus H.A., Schweizer P.A. (2019). ANK2 functionally interacts with KCNH2 aggravating long QT syndrome in a double mutation carrier. Biochem. Biophys. Res. Commun..

[13] Mesquita F.C.P., Arantes P.C., Kasai-Brunswick T.H., Araujo D.S., Gubert F., Monnerat G., Silva Dos Santos D., Neiman G., Leitao I.C., Barbosa R.A.Q. (2019). R534C mutation in hERG causes a trafficking defect in iPSC-derived cardiomyocytes from patients with type 2 long QT syndrome. Sci. Rep..

[14] Zahavich L., Tarnopolsky M., Yao R., Mital S. (2018). Novel Association of a De Novo CALM2 Mutation with Long QT Syndrome and Hypertrophic Cardiomyopathy. Circ. Genom. Precis. Med..

[15] Knight K.K., Olson D.R., Zhou R., Snyder P.M. (2006). Liddle’s syndrome mutations increase Na^+^ transport through dual effects on epithelial Na^+^ channel surface expression and proteolytic cleavage. Proc. Natl. Acad. Sci. USA.

[16] Rooj A.K., Cormet-Boyaka E., Clark E.B., Qadri Y.J., Lee W., Boddu R., Agarwal A., Tambi R., Uddin M., Parpura V. (2021). Association of cystic fibrosis transmembrane conductance regulator with epithelial sodium channel subunits carrying Liddle’s syndrome mutations. Am. J. Physiol. Lung Cell. Mol. Physiol..

[17] Rossi E., Farnetti E., Debonneville A., Nicoli D., Grasselli C., Regolisti G., Negro A., Perazzoli F., Casali B., Mantero F. (2008). Liddle’s syndrome caused by a novel missense mutation (P617L) of the epithelial sodium channel beta subunit. J. Hypertens..

[18] Staub O., Dho S., Henry P., Correa J., Ishikawa T., McGlade J., Rotin D. (1996). WW domains of Nedd4 bind to the proline-rich PY motifs in the epithelial Na+ channel deleted in Liddle’s syndrome. EMBO J..

[19] Ben-Ari J., Greenberg M., Nemet D., Edelstein E., Eliakim A. (2013). Octreotide-induced hepatitis in a child with persistent hyperinsulinemia hypoglycemia of infancy. J. Pediatr. Endocrinol. Metab..

[20] Gloyn A.L. (2003). Glucokinase (GCK) mutations in hyper- and hypoglycemia: Maturity-onset diabetes of the young, permanent neonatal diabetes, and hyperinsulinemia of infancy. Hum. Mutat..

[21] Hufnagel M., Eichmann D., Stieh J., Santer R. (1998). Further evidence for a dominant form of familial persistent hyperinsulinemic hypoglycemia of infancy: A family with documented hyperinsulinemia in two generations. J. Clin. Endocrinol. Metab..

[22] Phulwani P., Bergwitz C., Jaureguiberry G., Rasoulpour M., Estrada E. (2011). Hereditary hypophosphatemic rickets with hypercalciuria and nephrolithiasis-identification of a novel SLC34A3/NaPi-IIc mutation. Am. J. Med. Genet. A.

[23] Stapleton F.B. (1998). Making a “dent” in hereditary hypercalciuric nephrolithiasis. J. Pediatr..

[24] Tanaka K., Fisher S.E., Craig I.W. (1999). Characterization of novel promoter and enhancer elements of the mouse homologue of the Dent disease gene, CLCN5, implicated in X-linked hereditary nephrolithiasis. Genomics.

[25] Hudson A.J., Ebers G.C., Bulman D.E. (1995). The skeletal muscle sodium and chloride channel diseases. Brain.

[26] Chirasani V.R., Xu L., Addis H.G., Pasek D.A., Dokholyan N.V., Meissner G., Yamaguchi N. (2019). A central core disease mutation in the Ca(2+)-binding site of skeletal muscle ryanodine receptor impairs single-channel regulation. Am. J. Physiol. Cell Physiol..

[27] Denniss A., Dulhunty A.F., Beard N.A. (2018). Ryanodine receptor Ca(2+) release channel post-translational modification: Central player in cardiac and skeletal muscle disease. Int. J. Biochem. Cell Biol..

[28] Jung H.W., Kim K.I., Park C.G., Kang D.H., Ahn Y., Bae J.H., Kim C.H. (2015). A multicenter, non-comparative study to evaluate the efficacy and safety of fixed-dose olmesartan/amlodipine in Korean patients with hypertension who are naive or non-responders to anti-hypertensive monotherapy (ACE-HY study). Clin. Exp. Hypertens..

[29] Kuga K., Xu D.Z., Ohtsuka M., Aonuma K., Lau A.H., Watanabe Y., Ohtsuka K. (2011). Comparison of daily anti-hypertensive effects of amlodipine and nifedipine coat-core using ambulatory blood pressure monitoring-utility of "hypobaric curve" and "hypobaric area". Clin. Exp. Hypertens..

[30] Luo Y., Ren L., Jiang M., Chu Y. (2019). Anti-hypertensive efficacy of amlodipine dosing during morning versus evening: A meta-analysis. Rev. Cardiovasc. Med..

[31] Takenaka T., Uchida K., Kojima E., Gen S., Nodaira Y., Hoshi H., Kato N., Takane H., Ohno Y., Suzuki H. (2011). Amlodipine and loop diuretics as the second anti-hypertensive medication for the treatment of hypertension with chronic kidney diseases. Clin. Exp. Hypertens..

[32] Yousefpour A., Modarress H., Goharpey F., Amjad-Iranagh S. (2015). Interaction of PEGylated anti-hypertensive drugs, amlodipine, atenolol and lisinopril with lipid bilayer membrane: A molecular dynamics simulation study. Biochim. Biophys. Acta.

[33] Orubu E.S.F., Duncan J., Tuleu C., Turner M.A., Nunn A. (2021). WHO essential medicines for children 2011–2019: Age-appropriateness of enteral formulations. Arch. Dis. Child..

[34] Beeton C., Pennington M.W., Norton R.S. (2011). Analogs of the sea anemone potassium channel blocker ShK for the treatment of autoimmune diseases. Inflamm. Allergy Drug Targets.

[35] Chen R., Chung S.H. (2012). Engineering a potent and specific blocker of voltage-gated potassium channel Kv1.3, a target for autoimmune diseases. Biochemistry.

[36] Buono R.J., Lohoff F.W., Sander T., Sperling M.R., O’Connor M.J., Dlugos D.J., Ryan S.G., Golden G.T., Zhao H., Scattergood T.M. (2004). Association between variation in the human KCNJ10 potassium ion channel gene and seizure susceptibility. Epilepsy. Res..

[37] Calder J.A., Schachter M., Sever P.S. (1993). Ion channel involvement in the acute vascular effects of thiazide diuretics and related compounds. J. Pharmacol. Exp. Ther..

[38] Carter L.A., Belknap J.K., Crabbe J.C., Janowsky A. (1995). Allosteric regulation of the N-methyl-D-aspartate receptor-linked ion channel complex and effects of ethanol in ethanol-withdrawal seizure-prone and -resistant mice. J. Neurochem..

[39] Jones-Muhammad M., Shao Q., Cain-Shields L., Shaffery J.P., Warrington J.P. (2021). Acid Sensing Ion Channel 2a Is Reduced in the Reduced Uterine Perfusion Pressure Mouse Model and Increases Seizure Susceptibility in Pregnant Mice. Cells.

[40] Zhang H., Gao G., Zhang Y., Sun Y., Li H., Dong S., Ma W., Liu B., Wang W., Wu H. (2017). Glucose Deficiency Elevates Acid-Sensing Ion Channel 2a Expression and Increases Seizure Susceptibility in Temporal Lobe Epilepsy. Sci. Rep..

[41] Izumi-Nakaseko H., Hagiwara-Nagasawa M., Naito A.T., Goto A., Chiba K., Sekino Y., Kanda Y., Sugiyama A. (2018). Application of human induced pluripotent stem cell-derived cardiomyocytes sheets with microelectrode array system to estimate antiarrhythmic properties of multi-ion channel blockers. J. Pharmacol. Sci..

[42] Pugsley M.K., Hayes E.S., Saint D.A., Walker M.J.A. (2019). The antiarrhythmic actions of bisaramil and penticainide result from mixed cardiac ion channel blockade. Biomed. Pharmacother..

[43] Sterbuleac D., Maniu C.L. (2016). An antiarrhythmic agent as a promising lead compound for targeting the hEAG1 ion channel in cancer therapy: Insights from molecular dynamics simulations. Chem. Biol. Drug Des..

[44] Sterbuleac D., Maniu C.L. (2018). Computer Simulations Reveal a Novel Blocking Mode of the hERG Ion Channel by the Antiarrhythmic Agent Clofilium. Mol. Inform..

[45] Wang M., Shan J., Yang Q., Ma X., Jin S., Guo X., You Q., Tang Y. (2014). Antiarrhythmic efficacy of CPUY102122, a multiple ion channel blocker, on rabbits with ischemia/reperfusion injury. Pharmacol. Rep..

[46] Wu T., Nguyen H.X., Bursac N. (2021). In vitro discovery of novel prokaryotic ion channel candidates for antiarrhythmic gene therapy. Methods Enzymol..

[47] Kario K., Matsuda S., Nagahama S., Kurose Y., Sugii H., Teshima T., Suzuki N. (2021). Single-pill combination of cilnidipine, an l-/n-type calcium channel blocker, and valsartan reduces the day-by-day variability of morning home systolic blood pressure in patients with treated hypertension: A sub-analysis of the HOPE-combi survey. J. Clin. Hypertens..

[48] Miyoshi T., Onoue G., Ito H. (2019). Effect of Switching to Azilsartan From Fixed-Dose Combination of an Angiotensin II Receptor Blocker and Calcium Channel Blocker or a Thiazide in Patients With Hypertension. J. Clin. Med. Res..

[49] Garcia M.L., Kaczorowski G.J. (2016). Ion channels find a pathway for therapeutic success. Proc. Natl. Acad. Sci. USA.

[50] Veit G., Avramescu R.G., Chiang A.N., Houck S.A., Cai Z., Peters K.W., Hong J.S., Pollard H.B., Guggino W.B., Balch W.E. (2016). From CFTR biology toward combinatorial pharmacotherapy: Expanded classification of cystic fibrosis mutations. Mol. Biol. Cell.

[51] Latham J.L., Martin S.N. (2014). Infiltrative anesthesia in office practice. Am. Fam. Physician.

[52] Zhou D., Wang L., Cui Q., Iftikhar R., Xia Y., Xu P. (2020). Repositioning Lidocaine as an Anticancer Drug: The Role beyond Anesthesia. Front. Cell Dev. Biol..

[53] Sloop K.W., Emmerson P.J., Statnick M.A., Willard F.S. (2018). The current state of GPCR-based drug discovery to treat metabolic disease. Br. J. Pharmacol..

[54] Hauser A.S., Kooistra A.J., Munk C., Heydenreich F.M., Veprintsev D.B., Bouvier M., Babu M.M., Gloriam D.E. (2021). GPCR activation mechanisms across classes and macro/microscales. Nat. Struct. Mol. Biol..

[55] Dascal N. (2001). Ion-channel regulation by G proteins. Trends Endocrinol. Metab..

[56] Kottgen M., Benzing T., Simmen T., Tauber R., Buchholz B., Feliciangeli S., Huber T.B., Schermer B., Kramer-Zucker A., Hopker K. (2005). Trafficking of TRPP2 by PACS proteins represents a novel mechanism of ion channel regulation. EMBO J..

[57] Wickman K., Clapham D.E. (1995). Ion channel regulation by G proteins. Physiol. Rev..

[58] Zhang W. (2011). Roles of heterotrimeric G proteins in guard cell ion channel regulation. Plant Signal. Behav..

[59] Du Y., Duc N.M., Rasmussen S.G.F., Hilger D., Kubiak X., Wang L., Bohon J., Kim H.R., Wegrecki M., Asuru A. (2019). Assembly of a GPCR-G Protein Complex. Cell.

[60] Hauser A.S., Attwood M.M., Rask-Andersen M., Schioth H.B., Gloriam D.E. (2017). Trends in GPCR drug discovery: New agents, targets and indications. Nat. Rev. Drug Discov..

[61] Hauser A.S., Chavali S., Masuho I., Jahn L.J., Martemyanov K.A., Gloriam D.E., Babu M.M. (2018). Pharmacogenomics of GPCR Drug Targets. Cell.

[62] Pierce K.L., Premont R.T., Lefkowitz R.J. (2002). Seven-transmembrane receptors. Nat. Rev. Mol. Cell. Biol..

[63] Genomes Project C., Auton A., Brooks L.D., Durbin R.M., Garrison E.P., Kang H.M., Korbel J.O., Marchini J.L., McCarthy S., McVean G.A. (2015). A global reference for human genetic variation. Nature.

[64] Lakhan R., Kumari R., Misra U.K., Kalita J., Pradhan S., Mittal B. (2009). Differential role of sodium channels SCN1A and SCN2A gene polymorphisms with epilepsy and multiple drug resistance in the north Indian population. Br. J. Clin. Pharmacol..

[65] Jensen-Seaman M.I., Furey T.S., Payseur B.A., Lu Y., Roskin K.M., Chen C.F., Thomas M.A., Haussler D., Jacob H.J. (2004). Comparative recombination rates in the rat, mouse, and human genomes. Genome Res..

[66] Chimpanzee S., Analysis C. (2005). Initial sequence of the chimpanzee genome and comparison with the human genome. Nature.

[67] Nusbaum C., Mikkelsen T.S., Zody M.C., Asakawa S., Taudien S., Garber M., Kodira C.D., Schueler M.G., Shimizu A., Whittaker C.A. (2006). DNA sequence and analysis of human chromosome 8. Nature.

[68] Hellmann I., Prufer K., Ji H., Zody M.C., Paabo S., Ptak S.E. (2005). Why do human diversity levels vary at a megabase scale?. Genome Res..

[69] Lucas H.B., McKnight I., Raines R., Hijazi A., Hart C., Lee C., Kim D.G., Li W., Lee P.H.U., Shim J.W. (2021). Factors Associated with Mutations: Their Matching Rates to Cardiovascular and Neurological Diseases. Int. J. Mol. Sci..

[70] McKnight I., Hart C., Park I.H., Shim J.W. (2020). Genes causing congenital hydrocephalus: Their chromosomal characteristics of telomere proximity and DNA compositions. Exp. Neurol..

[71] Mallapaty S. (2021). Kids and COVID: Why young immune systems are still on top. Nature.

[72] Lopez Bernal J., Andrews N., Gower C., Gallagher E., Simmons R., Thelwall S., Stowe J., Tessier E., Groves N., Dabrera G. (2021). Effectiveness of COVID-19 Vaccines against the B.1.617.2 (Delta) Variant. N. Engl. J. Med..

[73] Cohen P., Cross D., Janne P.A. (2021). Kinase drug discovery 20 years after imatinib: Progress and future directions. Nat. Rev. Drug Discov..

[74] Hochstetler A.E., Smith H.M., Preston D.C., Reed M.M., Territo P.R., Shim J.W., Fulkerson D., Blazer-Yost B.L. (2020). TRPV4 antagonists ameliorate ventriculomegaly in a rat model of hydrocephalus. JCI Insight.

[75] Gruber C.N., Patel R.S., Trachtman R., Lepow L., Amanat F., Krammer F., Wilson K.M., Onel K., Geanon D., Tuballes K. (2020). Mapping Systemic Inflammation and Antibody Responses in Multisystem Inflammatory Syndrome in Children (MIS-C). Cell.

[76] Attwood M.M., Jonsson J., Rask-Andersen M., Schioth H.B. (2020). Soluble ligands as drug targets. Nat. Rev. Drug Discov..

[77] Shim J.W., Territo P.R., Simpson S., Watson J.C., Jiang L., Riley A.A., McCarthy B., Persohn S., Fulkerson D., Blazer-Yost B.L. (2019). Hydrocephalus in a rat model of Meckel Gruber syndrome with a TMEM67 mutation. Sci. Rep..

[78] Olkhovskiy I.A., Gorbenko A.S., Stolyar M.A., Grischenko D.A., Tkachenko O.A., Martsinkevich T.L. (2019). Somatic mutation of the V617F JAK2 gene in patients of the cardiovascular diseases. Ter. Arkh..

[79] Garrett-Bakelman F.E., Darshi M., Green S.J., Gur R.C., Lin L., Macias B.R., McKenna M.J., Meydan C., Mishra T., Nasrini J. (2019). The NASA Twins Study: A multidimensional analysis of a year-long human spaceflight. Science.

[80] Fuster J.J., Walsh K. (2018). Somatic Mutations and Clonal Hematopoiesis: Unexpected Potential New Drivers of Age-Related Cardiovascular Disease. Circ. Res..

[81] Sadhu B., Sundararajan M., Bandyopadhyay T. (2017). Divalent ions are potential permeating blockers of the non-selective NaK ion channel: Combined QM and MD based investigations. Phys. Chem. Chem. Phys..

[82] Norager N.G., Poulsen M.H., Stromgaard K. (2018). Controlling Ca(2+) Permeable alpha-Amino-3-hydroxy-5-methyl-4-isoxazolepropionic Acid (AMPA) Receptors with Photochromic Ion Channel Blockers. J. Med. Chem..

[83] Rotov A.Y., Astakhova L.A., Sitnikova V.S., Evdokimov A.A., Boitsov V.M., Dubina M.V., Ryazantsev M.N., Firsov M.L. (2018). New Experimental Models of Retinal Degeneration for Screening Molecular Photochromic Ion Channel Blockers. Acta Nat..

[84] Turman D.L., Cheloff A.Z., Corrado A.D., Nathanson J.T., Miller C. (2018). Molecular Interactions between a Fluoride Ion Channel and Synthetic Protein Blockers. Biochemistry.

[85] Xu R., Xiao Y., Liu Y., Wang B., Li X., Huo C., Jia X., Hou L., Wang X. (2018). Fluorescence-Based High Throughput Screening Technologies for Natural Chloride Ion Channel Blockers. Chem. Res. Toxicol..

[86] Liao Q., Feng Y., Yang B., Lee S.M. (2019). Cnidarian peptide neurotoxins: A new source of various ion channel modulators or blockers against central nervous systems disease. Drug Discov. Today.

[87] Khalifa N., Kumar Konda L.S., Kristam R. (2020). Machine learning-based QSAR models to predict sodium ion channel (Nav 1.5) blockers. Future Med. Chem..

[88] Kim T., Cho S., Oh H., Hur J., Kim H., Choi Y.H., Jeon S., Yang Y.D., Kim S.H. (2020). Design of Anticancer 2,4-Diaminopyrimidines as Novel Anoctamin 1 (ANO1) Ion Channel Blockers. Molecules.

[89] Wei F., Pourrier M., Strauss D.G., Stockbridge N., Pang L. (2020). Effects of Electrical Stimulation on hiPSC-CM Responses to Classic Ion Channel Blockers. Toxicol. Sci..

[90] Cholasseri R., De S. (2021). Dual-Site Binding of Quaternary Ammonium Ions as Internal K(+)-Ion Channel Blockers: Nonclassical (C-H…O) H Bonding vs. Dispersive (C-H…H-C) Interaction. J. Phys. Chem. B.

[91] Jaeschke H. (2021). Comments on “DNA-binding activities of compounds acting as enzyme inhibitors, ion channel blockers and receptor binders”. Chem. Biol. Interact..

[92] Mitini-Nkhoma S.C., Chimbayo E.T., Mzinza D.T., Mhango D.V., Chirambo A.P., Mandalasi C., Lakudzala A.E., Tembo D.L., Jambo K.C., Mwandumba H.C. (2021). Something Old, Something New: Ion Channel Blockers as Potential Anti-Tuberculosis Agents. Front. Immunol..

[93] Mosa F.E., Suryanarayanan C., Feng T., Barakat K. (2021). Effects of selective calcium channel blockers on ions’ permeation through the human Cav1.2 ion channel: A computational study. J. Mol. Graph. Model..

[94] Muhamedejevs R., Zivkovic L., Dzintare M., Sjakste N. (2021). DNA-binding activities of compounds acting as enzyme inhibitors, ion channel blockers and receptor binders. Chem. Biol. Interact..

[95] Sharonov G.V., Nekrasova O.V., Kudryashova K.S., Kirpichnikov M.P., Feofanov A.V. (2021). Bioengineered System for High Throughput Screening of Kv1 Ion Channel Blockers. Bioengineering.

[96] Zhang X., Johnson R.M., Drulyte I., Yu L., Kotecha A., Danev R., Wootten D., Sexton P.M., Belousoff M.J. (2021). Evolving cryo-EM structural approaches for GPCR drug discovery. Structure.

[97] Yanagawa M., Sako Y. (2021). Workflows of the Single-Molecule Imaging Analysis in Living Cells: Tutorial Guidance to the Measurement of the Drug Effects on a GPCR. Methods Mol. Biol..

[98] Woszczek G., Fuerst E., Maguire T.J.A. (2021). FLIPR Calcium Mobilization Assays in GPCR Drug Discovery. Methods Mol. Biol..

[99] Wang Y., Yu Z., Xiao W., Lu S., Zhang J. (2021). Allosteric binding sites at the receptor-lipid bilayer interface: Novel targets for GPCR drug discovery. Drug Discov. Today.

[100] Wang X., McFarland A., Madsen J.J., Aalo E., Ye L. (2021). The Potential of (19)F NMR Application in GPCR Biased Drug Discovery. Trends Pharmacol. Sci..

[101] Tian J.Y., Chi C.L., Bian G., Guo F.J., Wang X.Q., Yu B. (2021). A novel GPCR target in correlation with androgen deprivation therapy for prostate cancer drug discovery. Basic Clin. Pharmacol. Toxicol..

[102] Slosky L.M., Caron M.G., Barak L.S. (2021). Biased Allosteric Modulators: New Frontiers in GPCR Drug Discovery. Trends Pharmacol. Sci..

[103] Atkinson L.E., McCoy C.J., Crooks B.A., McKay F.M., McVeigh P., McKenzie D., Irvine A., Harrington J., Rosa B.A., Mitreva M. (2021). Phylum-Spanning Neuropeptide GPCR Identification and Prioritization: Shaping Drug Target Discovery Pipelines for Nematode Parasite Control. Front. Endocrinol..

[104] Wang P., Huang X., Qiu W., Xiao X. (2020). Identifying GPCR-drug interaction based on wordbook learning from sequences. BMC Bioinform..

[105] Salmaso V., Jacobson K.A. (2020). Purinergic Signaling: Impact of GPCR Structures on Rational Drug Design. ChemMedChem.

[106] Qiu W., Lv Z., Hong Y., Jia J., Xiao X. (2020). BOW-GBDT: A GBDT Classifier Combining With Artificial Neural Network for Identifying GPCR-Drug Interaction Based on Wordbook Learning From Sequences. Front. Cell Dev. Biol..

[107] Park J., Langmead C.J., Riddy D.M. (2020). New Advances in Targeting the Resolution of Inflammation: Implications for Specialized Pro-Resolving Mediator GPCR Drug Discovery. ACS Pharmacol. Transl. Sci..

[108] Mohammad Nezhady M.A., Rivera J.C., Chemtob S. (2020). Location Bias as Emerging Paradigm in GPCR Biology and Drug Discovery. iScience.

[109] Marti-Solano M., Crilly S.E., Malinverni D., Munk C., Harris M., Pearce A., Quon T., Mackenzie A.E., Wang X., Peng J. (2020). Combinatorial expression of GPCR isoforms affects signalling and drug responses. Nature.

[110] Marti-Solano M., Crilly S.E., Malinverni D., Munk C., Harris M., Pearce A., Quon T., Mackenzie A.E., Wang X., Peng J. (2020). Author Correction: Combinatorial expression of GPCR isoforms affects signalling and drug responses. Nature.

[111] Liu L., Jockers R. (2020). Structure-Based Virtual Screening Accelerates GPCR Drug Discovery. Trends Pharmacol. Sci..

[112] Hothersall J.D., Jones A.Y., Dafforn T.R., Perrior T., Chapman K.L. (2020). Releasing the technical ‘shackles’ on GPCR drug discovery: Opportunities enabled by detergent-free polymer lipid particle (PoLiPa) purification. Drug Discov. Today.

[113] Hatzipantelis C.J., Langiu M., Vandekolk T.H., Pierce T.L., Nithianantharajah J., Stewart G.D., Langmead C.J. (2020). Translation-Focused Approaches to GPCR Drug Discovery for Cognitive Impairments Associated with Schizophrenia. ACS Pharmacol. Transl. Sci..

[114] Congreve M., de Graaf C., Swain N.A., Tate C.G. (2020). Impact of GPCR Structures on Drug Discovery. Cell.

[115] Bondarev A.D., Attwood M.M., Jonsson J., Chubarev V.N., Tarasov V.V., Schioth H.B. (2020). Opportunities and challenges for drug discovery in modulating Adhesion G protein-coupled receptor (GPCR) functions. Expert Opin. Drug Discov..

[116] Zhou J., Wild C. (2019). GPCR Drug Discovery: Emerging Targets, Novel Approaches and Future Trends. Curr. Top Med. Chem..

[117] Zhao S., Wu B., Stevens R.C. (2019). Advancing Chemokine GPCR Structure Based Drug Discovery. Structure.

[118] Shimada I., Ueda T., Kofuku Y., Eddy M.T., Wuthrich K. (2019). GPCR drug discovery: Integrating solution NMR data with crystal and cryo-EM structures. Nat. Rev. Drug Discov..

[119] Reyes-Alcaraz A., Lee Y.N., Yun S., Hwang J.I., Seong J.Y. (2019). Monitoring GPCR-beta-arrestin1/2 Interactions in Real Time Living Systems to Accelerate Drug Discovery. J. Vis. Exp..

[120] Munk C., Mutt E., Isberg V., Nikolajsen L.F., Bibbe J.M., Flock T., Hanson M.A., Stevens R.C., Deupi X., Gloriam D.E. (2019). An online resource for GPCR structure determination and analysis. Nat. Methods.

[121] Insel P.A., Sriram K., Gorr M.W., Wiley S.Z., Michkov A., Salmeron C., Chinn A.M. (2019). GPCRomics: An Approach to Discover GPCR Drug Targets. Trends Pharmacol. Sci..

[122] Felder C.C. (2019). GPCR drug discovery-moving beyond the orthosteric to the allosteric domain. Adv. Pharmacol..

[123] Doijen J., Van Loy T., Landuyt B., Luyten W., Schols D., Schoofs L. (2019). Advantages and shortcomings of cell-based electrical impedance measurements as a GPCR drug discovery tool. Biosens. Bioelectron..

[124] Chan H.C.S., Li Y., Dahoun T., Vogel H., Yuan S. (2019). New Binding Sites, New Opportunities for GPCR Drug Discovery. Trends Biochem. Sci..

[125] Topiol S. (2018). Current and Future Challenges in GPCR Drug Discovery. Methods Mol. Biol..

[126] James T. (2018). Cheminformatics in the Service of GPCR Drug Discovery. Methods Mol. Biol..

[127] Hawkins P.C.D., Stahl G. (2018). Ligand-Based Methods in GPCR Computer-Aided Drug Design. Methods Mol. Biol..

[128] Ciancetta A., Jacobson K.A. (2018). Breakthrough in GPCR Crystallography and Its Impact on Computer-Aided Drug Design. Methods Mol. Biol..

[129] Vasudevan N.T. (2017). cAMP assays in GPCR drug discovery. Methods Cell Biol..

[130] Ma Q., Ye L., Liu H., Shi Y., Zhou N. (2017). An overview of Ca(2+) mobilization assays in GPCR drug discovery. Expert Opin. Drug Discov..

[131] Lutjens R., Rocher J.P. (2017). Recent advances in drug discovery of GPCR allosteric modulators for neurodegenerative disorders. Curr. Opin. Pharmacol..

[132] Irannejad R., Pessino V., Mika D., Huang B., Wedegaertner P.B., Conti M., von Zastrow M. (2017). Functional selectivity of GPCR-directed drug action through location bias. Nat. Chem. Biol..

[133] Chatenet D., Hebert T.E. (2017). Understanding GPCR signaling in the brain- the path to CNS drug discovery. Curr. Opin. Pharmacol..

[134] Vieira-Rocha M.S., Sousa J.B., Rodriguez-Rodriguez P., Morato M., Arribas S.M., Diniz C. (2020). Insights into sympathetic nervous system and GPCR interplay in fetal programming of hypertension: A bridge for new pharmacological strategies. Drug Discov. Today.

[135] Iyinikkel J., Murray F. (2018). GPCRs in pulmonary arterial hypertension: Tipping the balance. Br. J. Pharmacol..

[136] Tutunea-Fatan E., Caetano F.A., Gros R., Ferguson S.S. (2016). GRK2 targeted knock-down results in spontaneous hypertension, and altered vascular GPCR signaling. J. Biol. Chem..

[137] Sun G.C., Ho W.Y., Chen B.R., Cheng P.W., Cheng W.H., Hsu M.C., Yeh T.C., Hsiao M., Lu P.J., Tseng C.J. (2015). GPCR dimerization in brainstem nuclei contributes to the development of hypertension. Br. J. Pharmacol..

[138] Wright D.B., Tripathi S., Sikarwar A., Santosh K.T., Perez-Zoghbi J., Ojo O.O., Irechukwu N., Ward J.P., Schaafsma D. (2013). Regulation of GPCR-mediated smooth muscle contraction: Implications for asthma and pulmonary hypertension. Pulm. Pharmacol. Ther..

[139] Wang X., Bosonea A.M., Odenbach J., Fernandez-Patron C. (2012). Molecular Signals Elicited by GPCR Agonists in Hypertension, Cardiovascular Remodeling: Are MMPs and ADAMs Elusive Therapeutic Targets?. Curr. Hypertens. Rev..

[140] Nagareddy P.R., MacLeod K.M., McNeill J.H. (2010). GPCR agonist-induced transactivation of the EGFR upregulates MLC II expression and promotes hypertension in insulin-resistant rats. Cardiovasc. Res..

[141] Brinks H.L., Eckhart A.D. (2010). Regulation of GPCR signaling in hypertension. Biochim. Biophys. Acta.

[142] Harris D.M., Cohn H.I., Pesant S., Eckhart A.D. (2008). GPCR signalling in hypertension: Role of GRKs. Clin. Sci..

[143] Efendiev R., Krmar R.T., Ogimoto G., Zwiller J., Tripodi G., Katz A.I., Bianchi G., Pedemonte C.H., Bertorello A.M. (2004). Hypertension-linked mutation in the adducin alpha-subunit leads to higher AP2-mu2 phosphorylation and impaired Na^+^, K^+^-ATPase trafficking in response to GPCR signals and intracellular sodium. Circ. Res..

[144] Sushma, Mondal A.C. (2019). Role of GPCR signaling and calcium dysregulation in Alzheimer’s disease. Mol. Cell. Neurosci..

[145] Farfan-Garcia E.D., Marquez-Gomez R., Barron-Gonzalez M., Perez-Capistran T., Rosales-Hernandez M.C., Pinto-Almazan R., Soriano-Ursua M.A. (2019). Monoamines and their Derivatives on GPCRs: Potential Therapy for Alzheimer’s Disease. Curr. Alzheimer. Res..

[146] Haque M.E., Kim I.S., Jakaria M., Akther M., Choi D.K. (2018). Importance of GPCR-Mediated Microglial Activation in Alzheimer’s Disease. Front. Cell. Neurosci..

[147] Franco R., Martinez-Pinilla E., Navarro G., Zamarbide M. (2017). Potential of GPCRs to modulate MAPK and mTOR pathways in Alzheimer’s disease. Prog. Neurobiol..

[148] Zhao J., Deng Y., Jiang Z., Qing H. (2016). G Protein-Coupled Receptors (GPCRs) in Alzheimer’s Disease: A Focus on BACE1 Related GPCRs. Front. Aging Neurosci..

[149] Wentworth A.B., Hand J.L., Davis D.M., Tollefson M.M. (2021). Skin concerns in patients with trisomy 21 (Down syndrome): A Mayo Clinic 22-year retrospective review. Pediatr. Dermatol..

[150] Vazquez-Hernandez P.I., Cardenas-Conejo A., Catalan-Ruiz M.A., Navar-Gallegos K., Zenteno-Salazar E., Rafael-Parra-Bravo J., Aragon-Nogales R., Ibarra-Sarlat M., Nunez-Enriquez J.C. (2021). Multiple Organ Failure Associated with SARS-CoV-2 Infection in a Child with Down Syndrome: Is Trisomy 21 Associated with an Unfavourable Clinical Course?. Case Rep. Pediatr..

[151] Rafferty K., Archer K.J., Turner K., Brown R., Jackson-Cook C. (2021). Trisomy 21-associated increases in chromosomal instability are unmasked by comparing isogenic trisomic/disomic leukocytes from people with mosaic Down syndrome. PLoS ONE.

[152] Nguyen M., Litra F., Kamil A., Ergun-Longmire B. (2021). Intractable Vomiting in an 11-Month-Old Boy With Trisomy 21: A Case Report on Abnormal Calcium/Calcinosis/Creatinine in Down Syndrome. Cureus.

[153] Minter R., Gardiner K.J. (2021). Trisomy of Human Chromosome 21 Orthologs Mapping to Mouse Chromosome 10 Cause Age and Sex-Specific Learning Differences: Relevance to Down Syndrome. Genes.

[154] Huls A., Costa A.C.S., Dierssen M., Baksh R.A., Bargagna S., Baumer N.T., Brandao A.C., Carfi A., Carmona-Iragui M., Chicoine B.A. (2021). Medical vulnerability of individuals with Down syndrome to severe COVID-19-data from the Trisomy 21 Research Society and the UK ISARIC4C survey. EClinicalMedicine.

[155] Hasle H., Kline R.M., Kjeldsen E., Nik-Abdul-Rashid N.F., Bhojwani D., Verboon J.M., DiTroia S.P., Chao K.R., Raaschou-Jensen K., Palle J. (2021). Germline GATA1s generating mutations predispose to leukemia with acquired trisomy 21 and Down syndrome-like phenotype. Blood.

[156] Emes D., Huls A., Baumer N., Dierssen M., Puri S., Russell L., Sherman S.L., Strydom A., Bargagna S., Brandao A.C. (2021). COVID-19 in Children with Down Syndrome: Data from the Trisomy 21 Research Society Survey. J. Clin. Med..

[157] Cejas R.B., Tamano-Blanco M., Blanco J.G. (2021). Analysis of the intracellular traffic of IgG in the context of Down syndrome (trisomy 21). Sci. Rep..

[158] Schuster J., Hoeber J., Sobol M., Fatima A., Anneren G., Dahl N. (2020). Generation of two human iPSC lines (UUIGPi013-A and UUIPGi014-A) from cases with Down syndrome and full trisomy for chromosome 21 (T21). Stem Cell Res..

[159] McGregor-Schuerman M., Lo Fo Sang A., Bihari S., Ramdajal N., Suijkerbuijk R.F., van Ravenswaaij-Arts C.M. (2020). A child with complementary mosaic trisomy 8 and mosaic trisomy 21; clinical description of Warkany-Down syndrome and mechanism of origin. Eur. J. Med. Genet..

[160] Laurent A.P., Siret A., Ignacimouttou C., Panchal K., Diop M., Jenni S., Tsai Y.C., Roos-Weil D., Aid Z., Prade N. (2020). Constitutive Activation of RAS/MAPK Pathway Cooperates with Trisomy 21 and Is Therapeutically Exploitable in Down Syndrome B-cell Leukemia. Clin. Cancer Res..

[161] Czechowicz P., Malodobra-Mazur M., Lebioda A., Jonkisz A., Dobosz T., Smigiel R. (2020). Polymorphisms of the MTHFR gene in mothers of children with trisomy 21 (Down syndrome) in a Polish population. Adv. Clin. Exp. Med..

[162] Adams A.D., Guedj F., Bianchi D.W. (2020). Placental development and function in trisomy 21 and mouse models of Down syndrome: Clues for studying mechanisms underlying atypical development. Placenta.

[163] Pelleri M.C., Cicchini E., Petersen M.B., Tranebjaerg L., Mattina T., Magini P., Antonaros F., Caracausi M., Vitale L., Locatelli C. (2019). Partial trisomy 21 map: Ten cases further supporting the highly restricted Down syndrome critical region (HR-DSCR) on human chromosome 21. Mol. Genet. Genomic. Med..

[164] Levine M.S., Holland A.J. (2018). The impact of mitotic errors on cell proliferation and tumorigenesis. Genes. Dev..

[165] Pelleri M.C., Gennari E., Locatelli C., Piovesan A., Caracausi M., Antonaros F., Rocca A., Donati C.M., Conti L., Strippoli P. (2017). Genotype-phenotype correlation for congenital heart disease in Down syndrome through analysis of partial trisomy 21 cases. Genomics.

[166] Doran E., Keator D., Head E., Phelan M.J., Kim R., Totoiu M., Barrio J.R., Small G.W., Potkin S.G., Lott I.T. (2017). Down Syndrome, Partial Trisomy 21, and Absence of Alzheimer’s Disease: The Role of APP. J. Alzheimer’s Dis..

[167] Cardenas A.M., Fernandez-Olivares P., Diaz-Franulic I., Gonzalez-Jamett A.M., Shimahara T., Segura-Aguilar J., Caviedes R., Caviedes P. (2017). Knockdown of Myo-Inositol Transporter SMIT1 Normalizes Cholinergic and Glutamatergic Function in an Immortalized Cell Line Established from the Cerebral Cortex of a Trisomy 16 Fetal Mouse, an Animal Model of Human Trisomy 21 (Down Syndrome). Neurotox. Res..

[168] Aivazidis S., Coughlan C.M., Rauniyar A.K., Jiang H., Liggett L.A., Maclean K.N., Roede J.R. (2017). The burden of trisomy 21 disrupts the proteostasis network in Down syndrome. PLoS ONE.

[169] Su M.T., Kuan L.C., Chou Y.Y., Tan S.Y., Kuo T.C., Kuo P.L. (2016). Partial trisomy of chromosome 21 without the Down syndrome phenotype. Prenat. Diagn..

[170] Potter H. (2016). Beyond Trisomy 21: Phenotypic Variability in People with Down Syndrome Explained by Further Chromosome Mis-segregation and Mosaic Aneuploidy. J. Down Syndr. Chromosom. Abnorm..

[171] Pelleri M.C., Cicchini E., Locatelli C., Vitale L., Caracausi M., Piovesan A., Rocca A., Poletti G., Seri M., Strippoli P. (2016). Systematic reanalysis of partial trisomy 21 cases with or without Down syndrome suggests a small region on 21q22.13 as critical to the phenotype. Hum. Mol. Genet..

[172] Has R., Akel E.G., Kalelioglu I.H., Dural O., Yasa C., Esmer A.C., Yuksel A., Yildirim A., Ibrahimoglu L., Ermis H. (2016). Fetal nasal bone hypoplasia in the second trimester: Comparison of diagnostic methods for predicting trisomy 21 (Down syndrome). J. Clin. Ultrasound.

[173] Delabar J.M., Allinquant B., Bianchi D., Blumenthal T., Dekker A., Edgin J., O’Bryan J., Dierssen M., Potier M.C., Wiseman F. (2016). Changing Paradigms in Down Syndrome: The First International Conference of the Trisomy 21 Research Society. Mol. Syndromol..

[174] Caglayan E.S. (2016). Generation of improved human cerebral organoids from single copy DYRK1A knockout induced pluripotent stem cells in trisomy 21: Hypothetical solutions for neurodevelopmental models and therapeutic alternatives in down syndrome. Cell Biol. Int..

[175] Weitzdoerfer R., Toran N., Subramaniyan S., Pollak A., Dierssen M., Lubec G. (2015). A cluster of protein kinases and phosphatases modulated in fetal Down syndrome (trisomy 21) brain. Amino Acids.

[176] Iwarsson E., Kvist U., Hulten M.A. (2015). Disomy 21 in spermatozoa and the paternal origin of trisomy 21 Down syndrome. Mol. Cytogenet..

[177] Fausch C., Roosli C. (2015). The incudomalleolar articulation in Down syndrome (trisomy 21): A temporal bone study. Otol. Neurotol..

[178] Papoulidis I., Papageorgiou E., Siomou E., Oikonomidou E., Thomaidis L., Vetro A., Zuffardi O., Liehr T., Manolakos E., Vassilis P. (2014). A patient with partial trisomy 21 and 7q deletion expresses mild Down syndrome phenotype. Gene.

[179] Kamhieh-Milz J., Moftah R.F., Bal G., Futschik M., Sterzer V., Khorramshahi O., Burow M., Thiel G., Stuke-Sontheimer A., Chaoui R. (2014). Differentially expressed microRNAs in maternal plasma for the noninvasive prenatal diagnosis of Down syndrome (trisomy 21). Biomed. Res. Int..

[180] Hulten M.A., Oijerstedt L., Iwarsson E., Jonasson J. (2014). Maternal Germinal Trisomy 21 in Down Syndrome. J. Clin. Med..

[181] Hibaoui Y., Grad I., Letourneau A., Sailani M.R., Dahoun S., Santoni F.A., Gimelli S., Guipponi M., Pelte M.F., Bena F. (2014). Modelling and rescuing neurodevelopmental defect of Down syndrome using induced pluripotent stem cells from monozygotic twins discordant for trisomy 21. EMBO Mol. Med..

[182] Enea-Drapeau C., Huguet P., Carlier M. (2014). Misleading face-based judgment of cognitive level in intellectual disability: The case of trisomy 21 (Down syndrome). Res. Dev. Disabil..

[183] Capkova P., Misovicova N., Vrbicka D. (2014). Partial trisomy and tetrasomy of chromosome 21 without Down Syndrome phenotype and short overview of genotype-phenotype correlation. A case report. Biomed. Pap. Med. Fac. Univ. Palacky Olomouc. Czech Repub.

[184] Hastbacka J., de la Chapelle A., Kaitila I., Sistonen P., Weaver A., Lander E. (1992). Linkage disequilibrium mapping in isolated founder populations: Diastrophic dysplasia in Finland. Nat. Genet..

[185] Bagal S.K., Brown A.D., Cox P.J., Omoto K., Owen R.M., Pryde D.C., Sidders B., Skerratt S.E., Stevens E.B., Storer R.I. (2013). Ion channels as therapeutic targets: A drug discovery perspective. J. Med. Chem..

[186] Jeremic D., Sanchez-Rodriguez I., Jimenez-Diaz L., Navarro-Lopez J.D. (2021). Therapeutic potential of targeting G protein-gated inwardly rectifying potassium (GIRK) channels in the central nervous system. Pharmacol. Ther..

[187] Blednov Y.A., Stoffel M., Alva H., Harris R.A. (2003). A pervasive mechanism for analgesia: Activation of GIRK2 channels. Proc. Natl. Acad. Sci. USA.

[188] Duprat F., Lesage F., Patel A.J., Fink M., Romey G., Lazdunski M. (2000). The neuroprotective agent riluzole activates the two P domain K(+) channels TREK-1 and TRAAK. Mol. Pharmacol..

[189] Yang D., Zhou Q., Labroska V., Qin S., Darbalaei S., Wu Y., Yuliantie E., Xie L., Tao H., Cheng J. (2021). G protein-coupled receptors: Structure- and function-based drug discovery. Signal. Transduct. Target Ther..

[190] Imbrici P., Liantonio A., Camerino G.M., De Bellis M., Camerino C., Mele A., Giustino A., Pierno S., De Luca A., Tricarico D. (2016). Therapeutic Approaches to Genetic Ion Channelopathies and Perspectives in Drug Discovery. Front. Pharmacol..

