# Supplementary material for: Drug-Targeted Genomes: Mutability of Ion Channels and GPCRs
Source: Biomedicines. 2022 Mar 3;10(3):594. doi: 10.3390/biomedicines10030594 (PMC8945769; doi:10.3390/biomedicines10030594)
Supplement: Supplementary file 1 [file biomedicines-10-00594-s001.zip › biomedicines-1605330-supplementary.pdf]

Supplementary Materials

Drug targeted genomes: mutability of ion channels and GPCRs

Regan Raines[1], Ian McKnight[1], Hunter White[1], Kaitlyn Legg1[1], Chan Lee[2], Wei Li[3], Peter H.U. Lee[4,5], Joon W. Shim[1,*]

Correspondence to: shim@marshall.edu

This file includes:

Tables S1 to S3

Table S1. Two factor characteristics of 118 druggable ion channels

Ion channels	gene ID	chr*	gene locus	telomere locus	gene to telomere	A, T ** (%)	A + T (%)	FL*** (bp)
1	*HTR3B*	11	113 MB	135 Mb	135-113=22	26,27	26+27=53	4406
2	*HTR3E*	3	184 MB	198 Mb	198-184=14	23,25	23+25=48	1927
3	*HTR3D*	3	184 Mb	198 Mb	14	22,25	22+25=47	1459
4	*CHRNB2*	1	154 MB	248 MB	248-154=94	21,22	43	5857
5	*HTR3C*	3	184 MB	198 Mb	14	22,25	47	1699
6	*CHRND*	2	232 MB	242 Mb	10	21,23	44	2962
7	*CHRNB1*	17	7 Mb	83 Mb	76	22,25	27	2560
8	*CHRNA10*	10	3 Mb	133 MB	130	18,22	40	1945
9	*CHRNA2*	8	27 MB	145 Mb	118	19,24	43	4037
10	*CHRNA9*	4	40 MB	190 Mb	150	27,30	57	2272
11	*CHRNG*	2	232 Mb	242 Mb	10	23,25	48	4009
12	*ASIC4*	2	219 Mb	242 MB	23	19,19	38	2589
13	*ASIC5*	4	155 MB	189 Mb	34	29,32	61	1696
14	*BEST4*	1	44 Mb	248 Mb	204	19,24	43	2512
15	*CACNA2D1*	7	82 MB	159 MB	77	31,33	64	7542
16	*CACNA2D3*	3	54 Mb	198 Mb	144	27,25	52	3789
17	*CACNA2D4*	12	1.8 Mb	133 MB	131.2	24,23	47	5285
18	*CACNB1*	17	39 MB	81 Mb	42	20,21	41	3711
19	*CACNB2*	10	18 MB	131 MB	113	30,30	60	6199
20	*CACNB3*	12	48 MB	132 Mb	84	19,20	39	2931
21	*CACNA2D2*	3	50 MB	198 MB	148	22,21	43	5696
22	*CACNG6*	19	54 Mb	58 MB	4	15,22	37	2288
23	*CACNA1F*	X	49 MB	156 MB	107	21,24	45	6039
24	*CACNB4*	2	152 Mb	242 MB	90	32,34	66	7944
25	*CACNG1*	17	67 Mb	81 MB	14	18,23	41	1302
26	*CACNG5*	17	66 Mb	82 Mb	16	23,28	51	10576
27	*CACNG3*	16	24 Mb	90 Mb	66	24,25	49	1917
28	*CACNG7*	19	53 Mb	58 Mb	5	16,23	39	2754
29	*CACNG4*	17	67 MB	83 MB	16	20,22	42	3583
30	*CACNG8*	19	53 MB	58 MB	5	24,21	45	8850
31	*CLCNKA*	1	16 MB	248 Mb	232	19,23	42	2491
32	*CLIC6*	21	34 Mb	46 MB	12	24,21	45	4197
33	*CLIC2*	X	155 MB	156 Mb	1	35,30	65	2623
34	*CLCA4*	1	86 Mb	248 Mb	162	34,29	63	3211
35	*CLCC1*	1	108 MB	248 Mb	140	28,31	59	4683
36	*CLCA3P*	1	86 MB	248 Mb	162	34,32	66	4339
37	*CNGA4*	11	6.2 Mb	135 Mb	128.8	23,22	45	1916
38	*CLCN6*	1	11.8 Mb	248 Mb	236.2	21,27	48	5589
39	*CLIC5*	6	46 MB	169 Mb	123	28,28	56	5706
40	*CLCA2*	1	86 Mb	248 Mb	162	31,31	62	3935
41	*CLIC3*	9	136 Mb	138 MB	2	17,17	34	809
42	*CATSPER4*	1	26 Mb	248 Mb	222	23,23	46	1867
43	*CATSPER2*	15	43 Mb	101 Mb	58	27,30	57	4009
44	*FAM26F*	6	116 Mb	169 Mb	53	22,24	46	1127
45	*FAM26D*	6	116 Mb	169 Mb	53	22,24	46	1127

***** chr, chromosome; ** A, adenine; T, thymine; *** FL, full-length; bp, base pair

Table S1. Two factor characteristics of 118 druggable ion channels (continued)

**Ion channels**	gene ID	chr	gene locus	telomere locus	gene to telomere	A, T (%)	A + T (%)	FL (bp)
46	*FAM26E*	6	116 Mb	169 Mb	53	29,32	61	9787
47	*FXYD3*	19	35 Mb	58 MB	23	25,25	50	1378
48	*FXYD7*	19	35 Mb	58 MB	23	17,21	38	708
49	*FXYD6P3*	X	73 Mb	156 Mb	83	25,26	51	435
50	*GABRA6*	5	161 Mb	181 Mb	20	29,32	61	2450
51	*GABRG3*	15	27 MB	101 Mb	74	27,30	57	10862
52	*GABRR3*	3	98 MB	198 Mb	100	30,31	61	3113
53	*GABRB1*	4	47 MB	0	47	28,28	56	1939
54	*GABRG1*	4	46 MB	0	46	33,35	68	6758
55	*GABRP*	5	170 Mb	181 Mb	11	27,31	58	3304
56	*GABRA5*	15	26 Mb	101 Mb	75	29,27	56	2553
57	*GABRR1*	6	89 Mb	169 Mb	80	28,28	56	2742
58	*GPR89A*	1	145 Mb	248 Mb	103	26,31	57	2124
59	*GLRA3*	4	174 Mb	189 Mb	15	33,34	67	8697
60	*GLRA4*	X	103 Mb	155 Mb	52	23,29	52	3396
61	*GLRB*	4	157 Mb	189 Mb	32	30,34	64	3033
62	*GPR89A*	1	145 Mb	248 Mb	103	26,31	57	2124
63	*GRID1*	10	86 Mb	133 Mb	47	23,23	46	6154
64	*GRIK3*	1	36 Mb	0 Mb	36	23,22	45	9491
65	*HCN3*	1	155 Mb	248 MB	93	18,25	43	3838
66	*KCND1*	X	48 Mb	0	48	18,25	43	4718
67	*KCNK4*	11	64 Mb	134 Mb	70	14,20	34	1829
68	*KCNK7*	11	65 Mb	134 Mb	69	16,21	37	1386
69	*KCNS1*	20	45 MB	64 MB	19	23,24	47	5649
70	*KCNS2*	8	98 MB	144 Mb	46	23,29	52	5288
71	*KCNIP1*	5	170 Mb	181 Mb	11	24,24	48	2030
72	*KCNJ15*	21	38 MB	46 Mb	8	29,32	61	8399
73	*KCNMB4*	12	70 Mb	132 Mb	62	25,30	55	4717
74	*KCNK16*	12	132 Mb	132 Mb	0	23,24	47	3168
75	*ITPR2*	12	26 Mb	0	26	31,28	59	12564
76	*KCNH6*	17	63 Mb	82 Mb	19	20,20	40	3866
77	*KCNH7*	2	162 Mb	242 Mb	80	30,28	58	4262
78	*KCNH8*	8	19 Mb	0	19	28,29	57	5077
79	*KCNK12*	2	47 Mb	0	47	24,24	48	13564
80	*KCNMB3*	3	179 MB	197 Mb	18	29,33	62	6733
81	*KCNA6*	12	4 MB	0	4	24,20	44	3985
82	*KCNG2*	18	79 Mb	80 MB	1	11,18	29	1876
83	*KCNN1*	19	17 Mb	0	17	20,19	39	3633
84	*KCNAB2*	1	5 MB	0	5	19,21	40	3875
85	*KCNIP4*	4	20 Mb	0	20	32,31	63	2370
86	*KCNC4*	1	110 Mb	0	110	16,22	38	4829
87	*KCNG3*	2	42 Mb	0	42	24,30	54	3709
88	*KCNH4*	17	42 MB	82 Mb	40	18,21	39	3788
89	*KCNS3*	2	17 Mb	0	17	23,29	52	2341
90	*KCNAB3*	17	7 Mb	0	7	21,23	44	2879

Table S1. Two factor characteristics of 118 druggable ion channels (continued)

**Ion channels**	gene ID	chr	gene locus	telomere locus	gene to telomere	A, T (%)	A + T (%)	FL (bp)
91	*KCNG4*	16	84 Mb	90 Mb	6	21,25	46	5503
92	*KCNA7*	19	49 Mb	58 Mb	9	21,23	44	4151
93	*KCNT1*	9	135 Mb	138 MB	3	20,20	40	7123
94	*KCNJ14*	19	48 Mb	58 Mb	10	20,26	46	3854
95	*KCNJ18*	17	21 Mb	0	17	20,20	40	2196
96	*KCNV1*	8	109 MB	144 Mb	35	27,32	59	6912
97	*LRRC52*	1	165 MB	248 Mb	83	23,25	48	1372
98	*LRRC55*	11	57MB	134 Mb	77	26,26	52	5204
99	*LRRC38*	1	13 Mb	0	13	19,21	40	2168
100	*PANX3*	11	124 Mb	134 Mb	10	26,27	53	1729
101	*PANX2*	22	50 Mb	50.18	0.18	18,17	35	3052
102	*PLLP*	16	57 Mb	90 Mb	33	19,23	42	1497
103	*PKD1L2*	16	81 Mb	90 Mb	9	21,23	44	3451
104	*PKD2L2*	5	137 Mb	181 Mb	44	32,33	65	2214
105	*PKD1L3*	16	71 Mb	90 Mb	19	27,27	54	5623
106	*SLC26A1*	4	0.9 Mb	0	0.9	15,18	33	3413
107	*SCN3B*	11	123 Mb	134 MB	11	24,29	53	6082
108	*SCN7A*	2	166 Mb	240 Mb	74	30,34	64	7465
109	*SCNN1D*	1	1.2 Mb	0	1.2	18,18	36	3050
110	*SCN2B*	11	118 Mb	134 Mb	16	23,24	47	4937
111	*SCNN1B*	16	23 Mb	0	23	21,21	42	2560
112	*TMC5*	16	19 Mb	0	19	27,29	56	3676
113	*TMC7*	16	18 MB	0	18	26,29	55	4401
114	*TMEM38B*	9	105 MB	138 MB	33	30,35	65	3545
115	*TMC3*	15	81 Mb	101 Mb	20	25,26	51	4770
116	*TMC4*	19	54 Mb	58 Mb	4	16,25	41	2362
117	*TTYH2*	17	74 Mb	82 Mb	8	20,24	44	3433
118	*TTYH1*	19	54 Mb	58 Mb	4	16,22	38	2056

Table S2. Identification of 118 druggable ion channel types (30 anion channels in total)

Ion channels	gene ID	Gene name	Channel type
1	*HTR3B*	serotonin (5-HT) receptor 3B	cat- *
2	*HTR3E*	serotonin (5-HT) receptor 3E	cat-
3	*HTR3D*	serotonin (5-HT) receptor 3D	cat-
4	*CHRNB2*	instructor of neuronal nicotinic acetylcholine receptor (nAChR)	cat-
5	*HTR3C*	serotonin (5-HT) receptor 3C	cat-
6	*CHRND*	acetylcholine recep subunit delta	cat-
7	*CHRNB1*	acetylcholine recep subunit beta	cat-
8	*CHRNA10*	Neuronal acetylcholine receptor subunit alpha-10,	cat-
9	*CHRNA2*	instructor of neuronal nicotinic acetylcholine receptor (nAChR)	cat-
10	*CHRNA9*	cholinergic Receptor Nicotinic Alpha 9 Subunit	cat-
11	*CHRNG*	instructor of gamma protein component (subunit) of the actylcholine receptor (AChR)	cat-
12	*ASIC4*	Acid Sensing Ion Channel Subunit Family Member 4	Na^+
13	*ASIC5*	Acid Sensing Ion Channel Subunit Family Member 5	Na^+
14	*BEST4*	a member of the bestrophin gene family of anion channels	ani- **
15	*CACNA2D1*	Calcium Voltage-Gated Channel Auxiliary Subunit Alpha2delta 1	cat-
16	*CACNA2D3*	Calcium Voltage-Gated Channel Auxiliary Subunit Alpha2delta 3	cat-
17	*CACNA2D4*	Calcium Voltage-Gated Channel Auxiliary Subunit Alpha2delta 4	cat-
18	*CACNB1*	Voltage-dependent L-type calcium channel subunit beta-1	cat-
19	*CACNB2*	Voltage-dependent L-type calcium channel subunit beta-2	cat-
20	*CACNB3*	Voltage-dependent L-type calcium channel subunit beta-3	cat-
21	*CACNA2D2*	Calcium Voltage-Gated Channel Auxiliary Subunit Alpha2delta 2	cat-
22	*CACNG6*	Calcium Voltage-Gated Channel Auxiliary Subunit Gamma 6	cat-
23	*CACNA1F*	calcium Voltage-Gated Channel Subunit Alpha1 F	cat-
24	*CACNB4*	calcium Voltage-Gated Channel Auxiliary Subunit Beta 4	cat-
25	*CACNG1*	Voltage-dependent calcium channel gamma-1 subunit	cat-
26	*CACNG5*	Calcium Voltage-Gated Channel Auxiliary Subunit Gamma 5	cat-
27	*CACNG3*	Calcium Voltage-Gated Channel Auxiliary Subunit Gamma 3	cat-
28	*CACNG7*	Calcium Voltage-Gated Channel Auxiliary Subunit Gamma 7	cat-
29	*CACNG4*	Calcium Voltage-Gated Channel Auxiliary Subunit Gamma 4	cat-
30	*CACNG8*	Calcium Voltage-Gated Channel Auxiliary Subunit Gamma 8	cat-
31	*CLCNKA*	Chloride Voltage-Gated Channel Ka	Cl^-
32	*CLIC6*	Chloride Intracellular Channel 6	Cl^-
33	*CLIC2*	Chloride Intracellular Channel 2	Cl^-
34	*CLCA4*	chloride channel accessory 4,	Cl^-
35	*CLCC1*	chloride channel accessory 1	Cl^-
36	*CLCA3P*	Chloride Channel Accessory 3, Pseudogene	Cl^-
37	*CNGA4*	Cyclic Nucleotide Gated Channel Subunit Alpha 4	cat-
38	*CLCN6*	Cyclic Nucleotide Gated Channel Subunit Alpha 6	cat-
39	*CLIC5*	Chloride Intracellular Channel 5	Cl^-
40	*CLCA2*	Chloride channel accessory 2	Cl^-
41	*CLIC3*	chloride Intracellular Channel 3	Cl^-
42	*CATSPER4*	Cation Channel Sperm Associated 4	cat-
43	*CATSPER2*	Cation Channel Sperm Associated 2	cat-
44	*FAM26F*	Calcium Homeostasis Modulator Family Member 6	Ca^{2+}
45	*FAM26D*	Calcium Homeostasis Modulator Protein 4;	Ca^{2+}

** cat-, cation; ** ani-, anion; anion channels shaded in gray*

Table S2. Identification of 118 druggable ion channel types (continued)

Ion channels	gene ID	Gene name	Channel type
46	*FAM26E*	Calcium Homeostasis Modulator Family Member 5	Ca^{2+}
47	*FXYD3*	FXYD Domain Containing Ion Transport Regulator 3	cat-
48	*FXYD7*	FXYD Domain Containing Ion Transport Regulator 7	cat-
49	*FXYD6P3*	FXYD Domain Containing Ion Transport Regulator 6 Pseudogene 3	cat-
50	*GABRA6*	gamma-Aminobutyric Acid Type A Receptor Subunit Alpha6	Cl^-
51	*GABRG3*	Gamma-Aminobutyric Acid Type A Receptor Subunit Gamma3	Cl^-
52	*GABRR3*	Gamma-Aminobutyric Acid Type A Receptor Subunit Rho3	Cl^-
53	*GABRB1*	Gamma-Aminobutyric Acid Type A Receptor Subunit Beta1	Cl^-
54	*GABRG1*	Gamma-Aminobutyric Acid Type A Receptor Subunit Gamma1	Cl^-
55	*GABRP*	Gamma-Aminobutyric Acid Type A Receptor Subunit Pi	Cl^-
56	*GABRA5*	Gamma-Aminobutyric Acid Type A Receptor Subunit Alpha5	Cl^-
57	*GABRR1*	Gamma-aminobutyric acid receptor subunit rho-1	Cl^-
58	*GPR89A*	G Protein-Coupled Receptor 89A	ani-
59	*GLRA3*	Glycine Receptor Alpha 3	Cl^-
60	*GLRA4*	Glycine Receptor Alpha 4 (Pseudogene)	Cl^-
61	*GLRB*	Glycine receptor subunit beta	Cl^-
62	*GPR89A*	G Protein-Coupled Receptor 89A	ani-
63	*GRID1*	Glutamate Ionotropic Receptor Delta Type Subunit 1	cat-
64	*GRIK3*	Glutamate Ionotropic Receptor Kainate Type Subunit 3	cat-
65	*HCN3*	Potassium/sodium hyperpolarization-activated cyclic nucleotide-gated channel 3	K^+
66	*KCND1*	Potassium Voltage-Gated Channel Subfamily D Member 1	K^+
67	*KCNK4*	Potassium Two Pore Domain Channel Subfamily K Member 4	K^+
68	*KCNK7*	Potassium Two Pore Domain Channel Subfamily K Member 7	K^+
69	*KCNS1*	Potassium Voltage-Gated Channel Modifier Subfamily S Member 1	K^+
70	*KCNS2*	Potassium Voltage-Gated Channel Modifier Subfamily S Member 2	K^+
71	*KCNIP1*	Potassium Voltage-Gated Channel Interacting Protein 1	K^+
72	*KCNJ15*	Potassium Inwardly Rectifying Channel Subfamily J Member 15	K^+
73	*KCNMB4*	Potassium Calcium-Activated Channel Subfamily M Regulatory Beta Subunit 4	K^+
74	*KCNK16*	Potassium Two Pore Domain Channel Subfamily K Member 16	K^+
75	*ITPR2*	Inositol 1,4,5-Trisphosphate Receptor Type 2	Ca^{2+}
76	*KCNH6*	Potassium Voltage-Gated Channel Subfamily H Member 6	K^+
77	*KCNH7*	Potassium Voltage-Gated Channel Subfamily H Member 7	K^+
78	*KCNH8*	Potassium Voltage-Gated Channel Subfamily H Member 8	K^+
79	*KCNK12*	Potassium Two Pore Domain Channel Subfamily K Member 12	K^+
80	*KCNMB3*	Potassium Calcium-Activated Channel Subfamily M Regulatory Beta Subunit 3	K^+
81	*KCNA6*	Potassium Voltage-Gated Channel Subfamily A Member 6	K^+
82	*KCNG2*	Potassium Voltage-Gated Channel Modifier Subfamily G Member 2	K^+
83	*KCNN1*	Potassium Calcium-Activated Channel Subfamily N Member 1	K^+
84	*KCNAB2*	Potassium Voltage-Gated Channel Subfamily A Regulatory Beta Subunit 2	K^+
85	*KCNIP4*	Potassium Voltage-Gated Channel Interacting Protein 4	K^+
86	*KCNC4*	Potassium Voltage-Gated Channel Subfamily C Member 4	K^+
87	*KCNG3*	Potassium Voltage-Gated Channel Modifier Subfamily G Member 3	K^+
88	*KCNH4*	Potassium Voltage-Gated Channel Subfamily H Member 4	K^+
89	*KCNS3*	Potassium Voltage-Gated Channel Modifier Subfamily S Member 3	K^+
90	*KCNAB3*	Potassium Voltage-Gated Channel Subfamily A Regulatory Beta Subunit 3	Ca^{2+}

Table S2. Identification of 118 druggable ion channel types (continued)

**Ion channels**	gene ID	Gene name	Channel type
91	*KCNG4*	Potassium Voltage-Gated Channel Modifier Subfamily G Member 4	K^+
92	*KCNA7*	Potassium Voltage-Gated Channel Subfamily A Member 7	K^+
93	*KCNT1*	a sodium-activated potassium channel subunit	K^+
94	*KCNJ14*	Potassium Inwardly Rectifying Channel Subfamily J Member 14	K^+
95	*KCNJ18*	Potassium Inwardly Rectifying Channel Subfamily J Member 18	K^+
96	*KCNV1*	Potassium Voltage-Gated Channel Modifier Subfamily V Member 1	K^+
97	*LRRC52*	Leucine Rich Repeat Containing 52	K^+
98	*LRRC55*	Leucine Rich Repeat Containing 55	K^+
99	*LRRC38*	Leucine Rich Repeat Containing 38	K^+
100	*PANX3*	Pannexin 3	cat-
101	*PANX2*	Pannexin 2	cat-
102	*PLLP*	plasmolipin	K^+
103	*PKD1L2*	Polycystin 1 Like 2 (Gene/Pseudogene	cat-
104	*PKD2L2*	Polycystin 2 Like 2, Transient Receptor Potential Cation Channel	cat-
105	*PKD1L3*	Polycystin 1 Like 3, Transient Receptor Potential Channel Interacting	cat-
106	*SLC26A1*	Solute Carrier Family 26 Member 1	ani-
107	*SCN3B*	Sodium Voltage-Gated Channel Beta Subunit 3	Na^+
108	*SCN7A*	Sodium Voltage-Gated Channel Alpha Subunit 7	Na^+
109	*SCNN1D*	Sodium Channel Epithelial 1 Subunit Delta	Na^+
110	*SCN2B*	Sodium Voltage-Gated Channel Beta Subunit 2	Na^+
111	*SCNN1B*	the β subunit of the epithelial sodium channel ENaC	Na^+
112	*TMC5*	Transmembrane Channel Like 5	Cl^-
113	*TMC7*	Transmembrane Channel Like 7	Cl^-
114	*TMEM38B*	Transmembrane Protein 38B	cat-
115	*TMC3*	Transmembrane Channel Like 3	Cl^-
116	*TMC4*	Transmembrane Channel Like 4	Cl^-
117	*TTYH2*	Tweety Family Member 2	Cl^-
118	*TTYH1*	Tweety Family Member 1	Cl^-

Table S3. Two factor characteristics of 143 druggable GPCRs

GPCRs	gene ID	chr	gene locus	telomere locus	gene to telomere	A, T (%)	A + T (%)	FL (bp)
1	*HTR1E*	6	86 Mb	169 Mb	83	24,26	50	870
2	*HTR5A*	7	155 Mb	158 MB	3	26,29	55	924
3	*ADGRD1*	12	130 Mb	131 MB	1	21,25	46	924
4	*ADGRE2*	19	14 Mb	0	14	25,27	52	930
5	*ADGRF5*	6	46 Mb	0	46	29,28	57	944
6	*ADGRG4*	X	136 Mb	155 Mb	19	29,30	59	945
7	*ADGRG7*	3	100 Mb	197 Mb	97	30,31	61	954
8	*ADGRB2*	1	31 MB	0	31	16,20	36	957
9	*ADGRF2*	6	47 MB	0	47	27,31	58	960
10	*ADGRF4*	6	47 MB	0	47	27,29	56	997
11	*CHRM5*	15	33 Mb	0	33	28,28	56	1000
12	*ADGRF1*	6	47 MB	0	47	29,27	56	1000
13	*ADGRG3*	16	57 MB	0	57	19,24	43	1002
14	*ADGRG5*	16	57 MB	0	57	19,24	43	1017
15	*ADGRD2*	9	124 MB	0	124	15,19	34	1021
16	*ADGRE1*	19	6 MB	0	6	25,27	52	1027
17	*ADGRE3*	19	14 Mb	0	14	26,28	54	1033
18	*ADGRF3*	2	26 Mb	0	26	23,24	47	1043
19	*ADGRG2*	X	19 MB	0	19	27,31	58	1048
20	*ADGRB3*	6	68 MB	0	68	29,29	58	1060
21	*ADGRA1*	10	133 Mb	0	133	24,37	71	1074
22	*ADGRE4P*	19	6 Mb	0	6	26,28	54	1075
23	*ADGRL3*	4	61 MB	0	61	30,31	61	1078
24	*CELSR2*	1	109 Mb	0	109	18,22	40	1085
25	*GPR137*	11	64 Mb	0	64	14,23	37	1096
26	*FZD10*	12	130 Mb	0	130	20,23	43	1101
27	*GPR32P1*	19	50 MB	0	50	29,24	53	1137
28	*GALR3*	22	37 Mb	0	37	10,18	28	1147
29	*GPR139*	16	20 Mb	0	20	26,31	57	1151
30	*GPR149*	3	154 Mb	0	154	30,32	62	1198
31	*GPR171*	3	151 Mb	0	151	32,32	64	1236
32	*GPR174*	X	79 Mb	155 MB	76	28,36	64	1240
33	*GPR142*	17	74 Mb	82 Mb	8	18,22	40	1261
34	*GPR151*	5	146 Mb	181 Mb	35	29,32	61	1269
35	*GPR156*	3	120 Mb	0	120	23,25	48	1269
36	*GPRC5D*	12	12 MB	0	12	22,27	49	1289
37	*GPR153*	1	6 Mb	0	6	15,19	34	1311
38	*GPR33*	14	31 Mb	106 Mb	75	26,33	59	1347
39	*GPR25*	1	200 Mb	247 MB	47	9,19	28	1398
40	*GPR45*	2	105 MB	241 Mb	136	19,24	43	1408
41	*GPR75*	2	53 MB	0	53	23,29	52	1428
42	*GPR88*	1	100 Mb	0	100	22,27	49	1429
43	*GNRHR2*	1	145 MB	248 MB	103	22,26	48	1444
44	*GPR150*	5	95 Mb	181 Mb	86	14,23	37	1450
45	*GPR18*	13	99 Mb	114 Mb	15	29,31	60	1493
46	*GPR62*	3	51 Mb	0 Mb	51	16,20	36	1493
47	*GPR82*	X	41 MB	0	41	33,33	66	1564
48	*GPR87*	3	151 Mb	197	46	28,32	55	1564

Table S3. Two factor characteristics of 143 druggable GPCRs (continued)

GPCRs	gene ID	chr	gene locus	telomere locus	gene to telomere	A, T (%)	A + T (%)	FL (bp)
49	*GPR101*	X	137 MB	156 Mb	19	29,29	58	1571
50	*GPR173*	X	53 MB	0	53	20,24	44	1582
51	*GPRC5C*	17	74 Mb	83 MB	9	17,23	40	1586
52	*GPR32*	19	50 MB	58 MB	8	17,29	46	1626
53	*GPR63*	6	96 Mb	169 Mb	73	26,36	62	1637
54	*GPRC5B*	16	19 MB	0	19	23,27	50	1637
55	*GPR12*	13	26 MB	113 MB	87	27,32	59	1645
56	*GPR135*	14	59 Mb	106 Mb	47	26,28	54	1647
57	*GPR141*	7	37 Mb	0	37	29,35	64	1665
58	*GPR146*	7	1 Mb	0	1	20,22	42	1725
59	*GPR152*	11	67 MB	134 MB	67	15,21	36	1783
60	*GPR157*	1	9 MB	0	9	22,25	47	1788
61	*GPR160*	3	170 Mb	197 Mb	27	28,33	61	1810
62	*GPR42*	19	35 Mb	58 Mb	23	19,22	41	1815
63	*GPR52*	1	174 MB	248 Mb	74	24,34	58	1820
64	*GPR143*	X	9 Mb	0	9	20,26	46	1826
65	*GPR162*	12	6 Mb	0	6	19,20	39	1850
66	*GPR26*	10	123 Mb	133 Mb	10	25,27	52	1884
67	*GPR21*	9	123 MB	138 MB	15	21,32	53	1890
68	*GPR61*	1	109 Mb	0	109	23,27	50	1910
69	*GPR20*	8	141 Mb	144 Mb	3	16,21	37	1962
70	*GPR27*	3	71 Mb	0	71	18,26	44	2056
71	*GPR31*	6	167 Mb	170 Mb	3	22,29	51	2056
72	*GPR34*	X	41 Mb	0	41	33,33	66	2060
73	*GPR39*	2	132 Mb	242	110	22,24	46	2094
74	*GPR4*	10	45 Mb	135	90	20,24	44	2119
75	*GPR6*	6	109 Mb	171	62	18,24	42	2339
76	*GPR78*	4	8 Mb	0	8	18,24	42	2344
77	*GPR85*	7	113 Mb	159	46	28,31	59	2346
78	*GPR19*	12	12 Mb	0	12	27,32	59	2349
79	*GPR22*	7	107 Mb	159	52	35,32	67	2351
80	*HCAR1*	12	122 Mb	132	10	23,29	52	2417
81	*HCAR3*	12	122 Mb	132	10	23,27	50	2533
82	*MAS1L*	6	29 Mb	0	29	21,30	51	2576
83	*LPAR6*	13	48 Mb	114	66	31,32	63	2609
84	*MRGPRX4*	11	18 Mb	0	18	20,29	49	2642
85	*MRGPRG*	11	3 Mb	0	3	10,22	32	2709
86	*MRGPRX2*	11	19 Mb	0	19	23,32	55	2732
87	*MTNR1A*	4	186 Mb	190	4	19,23	42	2734
88	*MRGPRE*	11	3 Mb	0	3	18,20	38	2970
89	*MRGPRX3*	11	18 Mb	0	18	19,29	48	2988
90	*NPBWR1*	8	52 Mb	145	93	20,26	46	3005
91	*NPBWR2*	20	64 Mb	64	0	17,22	39	3047
92	*NPY6R*	5	137 Mb	181	44	30,30	60	3052
93	*NPY2R*	4	155 Mb	190	35	28,32	60	3112
94	*NPY5R*	4	163 Mb	190	27	29,34	63	3128
95	*OPN1MW2*	x	154 Mb	156	2	19,25	44	3128
96	*OXGR1*	13	97 Mb	114	17	27,32	59	3231

Table S3. Two factor characteristics of 143 druggable GPCRs (continued)

GPCRs	gene ID	chr	gene locus	telomere locus	gene to telomere	A, T (%)	A + T (%)	FL (bp)
97	*P2RY10*	X	79 Mb	156	77	29,34	63	3277
98	*OXER1*	2	42 Mb	0	42	16,22	38	3318
99	*RRH*	4	110 Mb	190	80	28,35	63	3323
100	*P2RY11*	19	10 Mb	0	10	18,17	35	3492
101	*PROKR1*	2	68 Mb	0	68	24,28	52	3517
102	*QRFPR*	4	121 Mb	190	69	26,26	52	3619
103	*GPRC5A*	12	13 Mb	0	13	21,31	52	3644
104	*RXFP3*	5	34 Mb	0	34	19,20	39	3752
105	*RXFP4*	1	156 Mb	248	92	16,25	41	3807
106	*S1PR4*	19	3 Mb	0	3	13,20	33	3837
107	*SSTR4*	20	23 Mb	0	23	21,24	45	3926
108	*SUCNR1*	3	152 Mb	198	46	31,35	66	4088
109	*TAS2R31*	12	11 Mb	0	11	26,35	61	4108
110	*TAS2R14*	12	11 Mb	0	11	26,35	61	4181
111	*TAAR3*	6	132 Mb	171	39	26,34	60	4198
112	*TAS2R10*	12	10 Mb	0	10	29,36	65	4209
113	*TAS2R41*	7	143 Mb	159	16	19,32	51	4250
114	*TAS2R43*	12	11 Mb	0	11	27,35	62	4287
115	*TAS2R50*	12	10 Mb	0	10	26,36	62	4343
116	*TAAR2*	6	132 Mb	171	39	25,36	61	4555
117	*TAAR8*	6	132 Mb	171	39	24,36	60	4573
118	*TAS2R19*	12	11 Mb	0	11	26,35	61	4622
119	*TAS2R7*	12	10 Mb	0	10	28,33	61	4675
120	*TAS2R9*	12	10 Mb	0	10	27,34	61	4769
121	*TAS2R13*	12	10 Mb	0	10	31,34	65	4787
122	*TAS2R20*	12	10 Mb	0	10	29,36	65	4832
123	*TAS2R40*	7	143 Mb	159	16	24,29	53	4851
124	*TAS2R60*	7	143 Mb	159	16	21,32	53	5018
125	*TAS2R3*	7	141 Mb	159	18	23,32	55	5026
126	*TAS2R4*	7	141 Mb	159	18	28,34	62	5144
127	*TAS2R5*	7	141 Mb	159	18	21,32	53	5197
128	*TAS2R16*	7	122 Mb	159	37	25,34	59	5241
129	*TAS2R8*	12	10 Mb	0	10	31,34	65	5390
130	*TAAR9*	6	132 Mb	171	39	25,34	59	5494
131	*TAS2R39*	7	143 MB	158 MB	15	24,33	57	5527
132	*TAS2R1*	5	9 MB	0	9	27,34	61	5705
133	*TAS2R30*	12	11 Mb	0	11	25,36	61	5741
134	*TAS2R42*	12	11 Mb	0	11	26,36	62	5802
135	*TAS2R46*	12	11 Mb	0	11	27,35	62	5967
136	*TPRA1*	3	127 MB	197 MB	70	20,25	45	6090
137	*AVPR1B*	1	206 MB	248 MB	42	22,25	47	6582
138	*VN1R17P*	1	247 MB	248 MB	1	no exon	no exon	6791
139	*VN1R3*	16	31 Mb	0	31	25,33	58	8032
140	*VN1R5*	1	247 MB	248 MB	1	24,34	58	9933
141	*VN1R1*	19	57 MB	58 MB	1	27,33	60	10306
142	*VN1R4*	19	53 MB	58 MB	5	22,31	53	11015
143	*VN1R2*	19	53 MB	58 MB	5	23,32	55	13516
